# Studies of Malagasy *Eugenia* – IV: Seventeen new endemic species, a new combination, and three lectotypifications; with comments on distribution, ecological and evolutionary patterns

**DOI:** 10.3897/phytokeys.49.9003

**Published:** 2015-04-28

**Authors:** Neil Snow, Martin Callmander, Peter B. Phillipson

**Affiliations:** 1 T.M. Sperry Herbarium, Department of Biology, Pittsburg State University, 1701 S. Broadway, Pittsburg, KS 66762 USA; 2 Missouri Botanical Garden, P.O. Box 299, St. Louis, MO 63166-0299, USA; 3 Conservatoire et Jardin botaniques de la Ville de Genève, case postale 60, 1292 Chambésy, Switzerland; 4 Institut de systématique, évolution, et biodiversité (ISYEB), Unité mixte de recherche 7205, Centre national de la recherche scientifique/Muséum national d’Histoire Naturelle/École pratique des hautes études, Université Pierre et Marie Curie, Sorbonne Universités, CP 39, 57 rue Cuvier, F-75231 Paris cedex 05, France

**Keywords:** Biogeography, conservation, *Eugenia*, *Eulemur fulvus*, leaf cutter bees, Madagascar, Myrtaceae, new species, systematics, vernacular names

## Abstract

Seventeen new endemic species of the genus *Eugenia* L. (Myrtaceae) are proposed from Madagascar, including: *Eugenia andapae* N. Snow, *Eugenia barriei* N. Snow, *Eugenia bemangidiensis* N. Snow, *Eugenia calciscopulorum* N. Snow, *Eugenia delicatissima* N. Snow, Callm. & Phillipson, *Eugenia echinulata* N. Snow, *Eugenia gandhii* N. Snow, *Eugenia hazonjia* N. Snow, *Eugenia iantarensis* N. Snow, *Eugenia malcomberi* N. Snow, *Eugenia manomboensis* N. Snow, *Eugenia obovatifolia* N. Snow, *Eugenia ranomafana* N. Snow & D. Turk, *Eugenia ravelonarivoi* N. Snow & Callm., *Eugenia razakamalalae* N. Snow & Callm., *Eugenia tiampoka* N. Snow & Callm., and *Eugenia wilsoniana* N. Snow, and one new combination, *Eugenia richardii* (Blume) N. Snow, Callm. & Phillipson is provided. Detailed descriptions, information on distribution and ecology, distribution maps, vernacular names (where known), digital images of types, comparisons to morphologically similar species. Preliminary assessment of IUCN risk of extinction and conservation recommendations are provided, including Vulnerable (4 species), Endangered (2 species), and Critically Endangered (4 species). Lectotpyes are designated for *Eugenia hovarum* H. Perrier, *Eugenia nompa* H. Perrier, and *Eugenia scottii* H. Perrier respectively.

## Abstract (fr)

Dix-sept nouvelles espèces endémiques du genre *Eugenia* L. (Myrtaceae) sont proposées pour Madagascar, incluant *Eugenia andapae* N. Snow, *Eugenia barriei* N. Snow, *Eugenia bemangidiensis* N. Snow, *Eugenia calciscopulorum* N. Snow, *Eugenia delicatissima* N. Snow, Callm. & Phillipson, *Eugenia echinulata* N. Snow, *Eugenia gandhii* N. Snow, *Eugenia hazonjia* N. Snow, *Eugenia iantarensis* N. Snow, *Eugenia malcomberi* N. Snow, *Eugenia manomboensis* N. Snow, *Eugenia obovatifolia* N. Snow, *Eugenia ranomafana* N. Snow & D. Turk, *Eugenia ravelonarivoi* N. Snow & Callm., *Eugenia razakamalalae* N. Snow & Callm., *Eugenia tiampoka* N. Snow & Callm., et *Eugenia wilsoniana* N. Snow, ainsi qu’une nouvelle combination, *Eugenia richardii* (Blume) N. Snow, Callm. & Phillipson. Des descriptions détaillées, des informations sur la distribution et l’écologie, des cartes de distribution, des noms vernaculaires (quand ils sont connus), des images digitalisées des types, des comparaisons avec les espèces morphologiquemnet proches et une évaluation préliminaire du risque d’extinction selon l’UICN ainsi que des recommendations de conservation sont fournies. Des lectotypes sont désignés pour *Eugenia hovarum* H. Perrier, *Eugenia nompa* H. Perrier et *Eugenia scottii* H. Perrier.

## Introduction

This paper is the fourth in a series devoted to clarifying the systematics of Malagasy *Eugenia* L. (Snow 2008, 2011; Snow et al. 2012), which until the work of Miller (2000) and Labat and Schatz (2002) had been dormant for five decades since early treatments by Perrier de la Bâthie (1953a,b). Its purpose is to propose seventeen new species, make a new combination, and lectotypify three names. It also provides detailed species descriptions with comparisons to morphologically similar taxa, distribution maps, vernacular names, digital images of types, and preliminary conservation assessments following IUCN Red List Categories and Criteria (2012).

## Materials and methods

Type specimens of all nearly described species of *Eugenia* from Madagascar have been examined. All Malagasy specimens of *Eugenia* housed at MO (abbreviations following Thiers (2015)) have been viewed by NS (through May 2014), as have all duplicates housed at his institution (KSP). Specimens housed at G and P have been studied by MC and PP. Duplicates of many specimens housed at KSP also have been consulted by NS. Specimens cited here also were compared against all material of *Eugenia* from the Comoros and Mascarenes housed at MO through May 2014. All specimens housed at MO should have duplicates at P and TAN given existing exchange agreements, although none at TAN have been seen by the authors. Accession numbers of specimens (where indicated) follow the herbarium acronym with a hyphen, whereas barcode numbers are enclosed in square brackets.

Species descriptions include all information that can be interpreted with confidence and, given available information, are more or less in parallel. The species concept and criteria follow previous applications (Snow 1997; Snow et al. 2003). Terminology largely follows earlier works for Malagasy *Eugenia* (e.g., Snow et al. 2012) but with greater consistency regarding general terminology (Beentjie 2010). Terms specific to Myrtaceae have been minimized, but may include anthopodium, metaxyphyll, brachyblast, bracteole, monad, and triad, given their descriptive utility in Myrtaceae (see Briggs and Johnson 1979). The collection number cited for each specimen follows immediately after the senior collector, in accordance with most database systems now in use. Geocoordinates in square brackets were determined retrospectively by various workers.

Hotlinks to holotypes on Tropicos® are included in lieu of illustrations of the new species. In addition, scanned images for all barcoded specimens from P are available online through the MNHN Vascular Plant Database (http://science.mnhn.fr/institution/mnhn/collection/p/item/search/form). Photos of living material are included when available. The conservation status of each species was assessed following IUCN Red List Categories and Criteria (IUCN 2012). The calculations of Area of Occupancy (AOO), Extent of Occurrence (EOO) and number of subpopulations follow the methodology of Callmander et al. (2007), using a grid cell size to calculate AOO of 3 × 3 km.

## Results and taxonomic treatments

### Eugenia andapae

Taxon classificationPlantaeMyrtalesMyrtaceae

**Figure 1.**
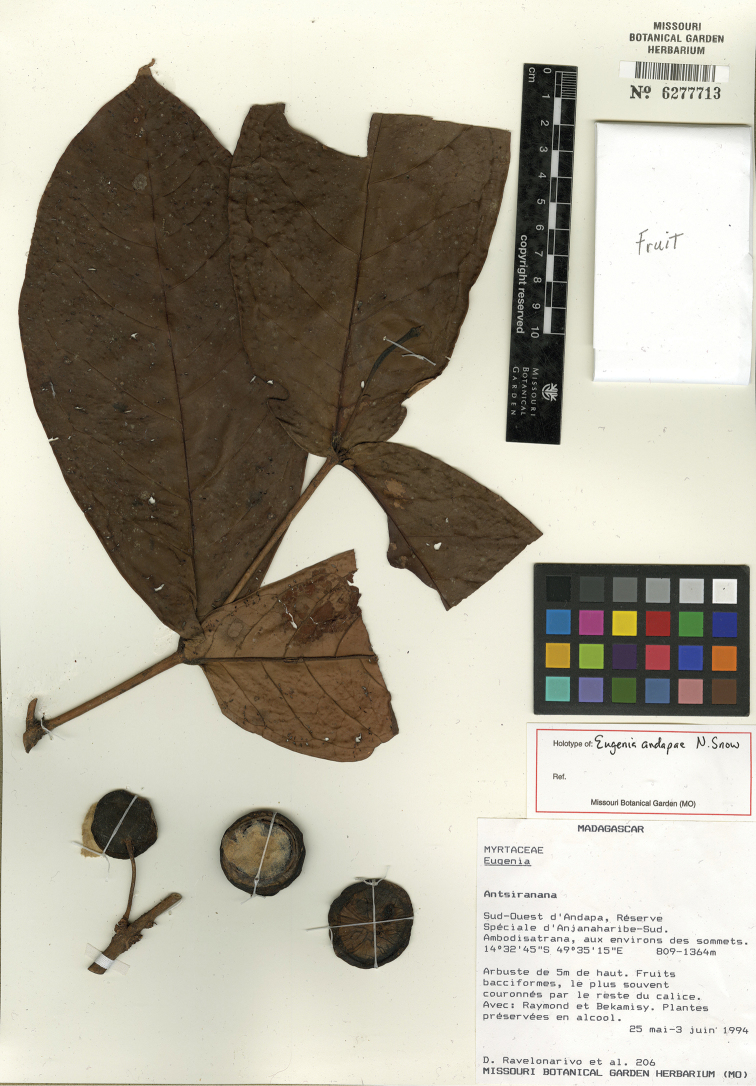
Holotype specimen of *Eugenia andapae* (MO).

N. Snow sp. nov.

urn:lsid:ipni.org:names:77146886-1

holotype (Figure 1): http://www.tropicos.org/Image/100314911

Frutex usque ad 9 m altus; ramuli complanati, glabri; folia usque ad 19 × 11.5 cm, obovata vel late elliptica, basi cordata vel rotundata, nervo medio supra sulcato, nervis secundariis infernis elevatis.

#### Type.

Madagascar. Prov. Antsiranana: Sud-Ouest d’Andapa, Réserve Spéciale d’Anjanaharibe-Sud. Ambodisatrana, aux environs des sommets, 14°32'45"S, 49°35'15"E, 809–1364 m, 25 May–3 June 1994, D. Ravelonarivo 206 + Raymond & Bekamisy (holotype: MO-6277713!; isotypes: KSP [KSP000041]!, P [P05097480]!, TAN).

#### Description.

Shrubs or trees 4–12 m tall; bark of main bole unknown. Vegetative and reproductive parts mostly glabrous except as noted. Branchlets laterally compressed, the terminal internode sometimes with a distal sulcus but becoming rounded, smooth, minutely and sparsely short-sericeous but soon glabrous, oil glands faint and moderately common but soon fading; emerging (youngest) internodes sometimes bearing 1–2 pairs of opposite to broadly ovate bracts 1.5–4.0 mm long. Leaves opposite, thinly coriaceous (dried material cracking with only moderate pressure), discolorous, matte above and below, venation brochidodromous. Axillary colleters absent. Petioles 3–4 mm long, slightly striate below, flattened above, epunctate. Leaf blades (15–)18.5–25 × (7–)13.5–14 cm, broadly elliptic to broadly obovate, base cordate and somewhat clasping (or rounded), surface and margin flat, apex obtuse to broadly acute, tip acuminate (or rarely retuse), secondary veins more or less straight, 11–15 per side protruding prominently below and arising at 25–55° angles and connected by slightly arching (but also prominent) inner marginal veins, tertiary veins relatively well-spaced but projecting only slightly (dried material); adaxial surface glabrous, oil glands faint (use magnification), sparse to common, more or less flush and darkish (dried), midvein sulcate; abaxial surface glabrous, midvein projecting prominently throughout and punctate (especially proximally) or epunctate, secondary veins projecting prominently, straight or only curving slightly towards margin, the secondaries connected at their ends by moderately arching connecting veins, tertiary veins projecting but less so than secondaries, intramarginal vein of same thickness as tertiaries, 1.5–5 mm from margin at midpoint of blade. Inflorescence (material scant) a monad; flowers cauliflorous, arising from short brachyblasts (< 3 mm long) above nodes on naked branches. Pedicels 8–32 mm long (possibly elongating after fertilization), 0.7–2.0 mm wide, somewhat compressed laterally, longitudinally striate, somewhat flexuous (bending with light touch), habit unknown, moderately glandular (glands faint), anthopodium and metaxyphylls absent. Bracteoles narrowly to broadly ovate, 1.5–2.5 × 0.5–1 mm, minutely and sparsely hairy dorsally and apically (hairs clear or whitish with some reddish). Hypanthium campanulate, 3.0–3.3 mm long, 3–4 mm wide at base of calyx lobes, densely but very shortly sericeous in proximal half (hairs reddish-brown) but glabrous distally; ovary apex glabrous. Calyx lobes 4 and often tearing irregularly towards hypanthium, up to 3.5 mm long × 4.5 mm broad (at base), irregularly hemispherical, glabrous on both faces apart from occasional minute hairs, evidently reflexed irregularly in anthesis. Petals 4 (material scant), 5.5–19.0 × ca. 10 mm, narrowly to broadly obovate, glabrous, epunctate, rose to violet. Staminal ring 3.5–4.0 mm in diameter (rounded or somewhat squarish), sparsely short hairy (hairs whitish). Stamens ca. 140 (estimated from scars on ring), multiseriate; filaments up to 10 mm long; anthers globular, ca. 0.8 mm long. Fruit 23–35 × 21–50 mm, depressed globular to globose, glabrous, base and apex rounded or apex crowned by calyx lobes, pinkish-reddish.

**Figure 2.**
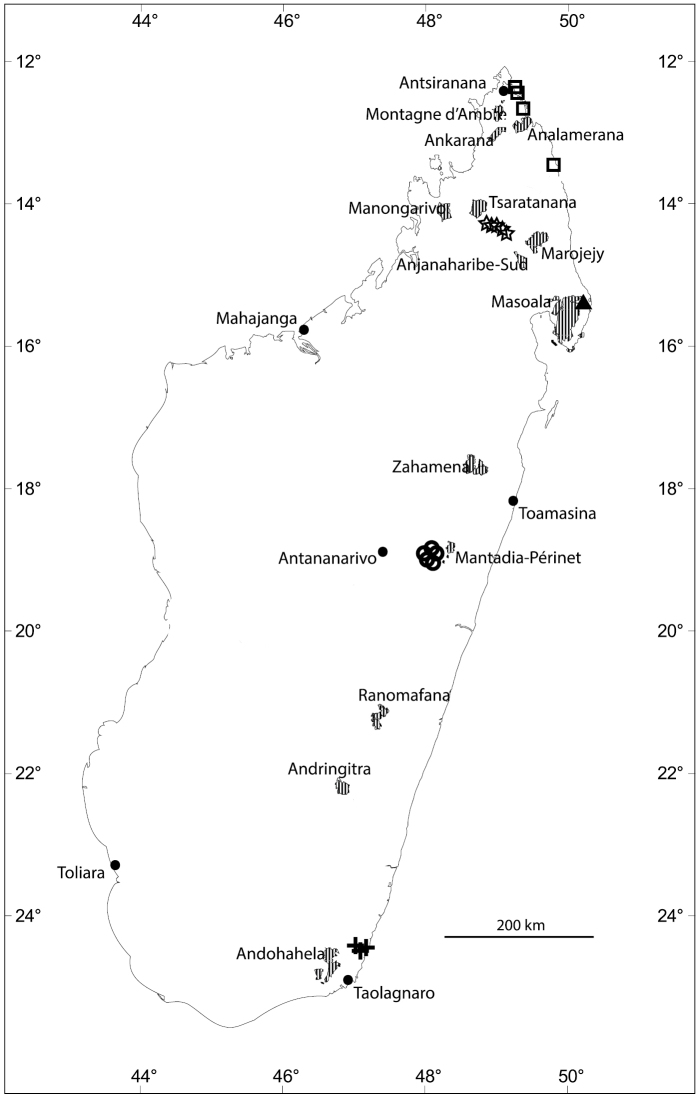
Distribution of new *Eugenia* species in Madagascar with selected Protected Areas (hatched): *Eugenia bemangidiensis* (crosses), *Eugenia razakamalalae* (triangle), *Eugenia richardii* (squares), *Eugenia tiampoka* (stars), and *Eugenia wilsoniana* (circles).

#### Phenology.

Flowering in February and March; fruiting March through November.

#### Etymology.

The species occurs in the Andapa Basin.

#### Distribution.

Known in northeast Madagascar in and around the Anjarahabe-Sud and Marojejy protected areas (Figure 7).

#### Habitat and ecology.

Humid forests, riparian areas and near summit of Ambodisatrana; ca. 200–1540 m.

#### Conservation status.

With an Extent of Occurrence (EOO) of 1586 km^2^, and Area of Occupancy (AOO) of 45 km^2^ and five subpopulations, two of which are situated within the protected area network (Anjanaharibe-Sud, Marojejy), *Eugenia andapae* is assigned a preliminary risk of extinction of “Vulnerable” [VU B1ab(iii)+2ab(iii)] following the IUCN Red List Categories and Criteria (IUCN 2012).

#### Comments.

The calyx lobes of this species tear towards the hypanthium during anthesis (see also *Eugenia lacerosepala* N. Snow and *Eugenia ambanizanensis* N. Snow). In this regard *Eugenia andapae* is similar to taxa first described by Scott (1979) from the Mascarenes in *Monimiastrum* A.J. Scott, which were reduced to synonymy under *Eugenia* (Snow 2008). The relatively long and broadly elliptic to obovate leaf blades with cordate bases, and the relatively straight and projecting abaxial secondary veins are diagnostic among other species of *Eugenia* in Madagascar. On herbarium material the abaxial tertiary veins also protrude slightly despite being thin. The label of the type specimen indicates that the fleshy fruits typically are crowned by the calyx lobes, although this was not true for the specimen from the Beamalona River.

#### Specimens examined.

MADAGASCAR. Prov. Antsiranana: Vallée inférieure de l’Androranga, affluent de la Bemarivo (NE), aux environs d’Antongondriha, à la base du massif du Betsomanga, [14°15'30"S, 49°44'00"E], 200 m, 17-20 Nov. 1950, H. Humbert 24234 + R. Capuron (P [P05208578]); Quartier d’Ambodisatrana, SW d’Andapa, Réserve Spéciale d’Anjanaharibe-Sud, suivant la piste au bord de la rivière de Beamalona, vers la chaîne d’Anjanaharibe dans la réserve, 14°38'30"S, 49°25'30"E, 1235 m, 23 Mar. 1995, D. Ravelonarivo 694 + R. Rabesonina (MO). Andapa, Anjialavabe, Ankiakabe, 14°09'50"S, 49°22'47"E, 952 m, 11 Feb. 2007, R. Razakamalala 3234 + D. Ravelonarivo, C. Rakotovao, Jacky & José (G, K, MO-6175410, P [P04885355]). Prov. Mahajanga: Amparihy, Ruisseau d’Andasinanantsomanga, 14°55'38"S, 49°25'50"E, 1199 m, 23 Feb. 2008, P. Bernard 860 + J. Ramiadana & J. Jocelyn (MO-6432613).

### Eugenia barriei

Taxon classificationPlantaeMyrtalesMyrtaceae

**Figure 3.**
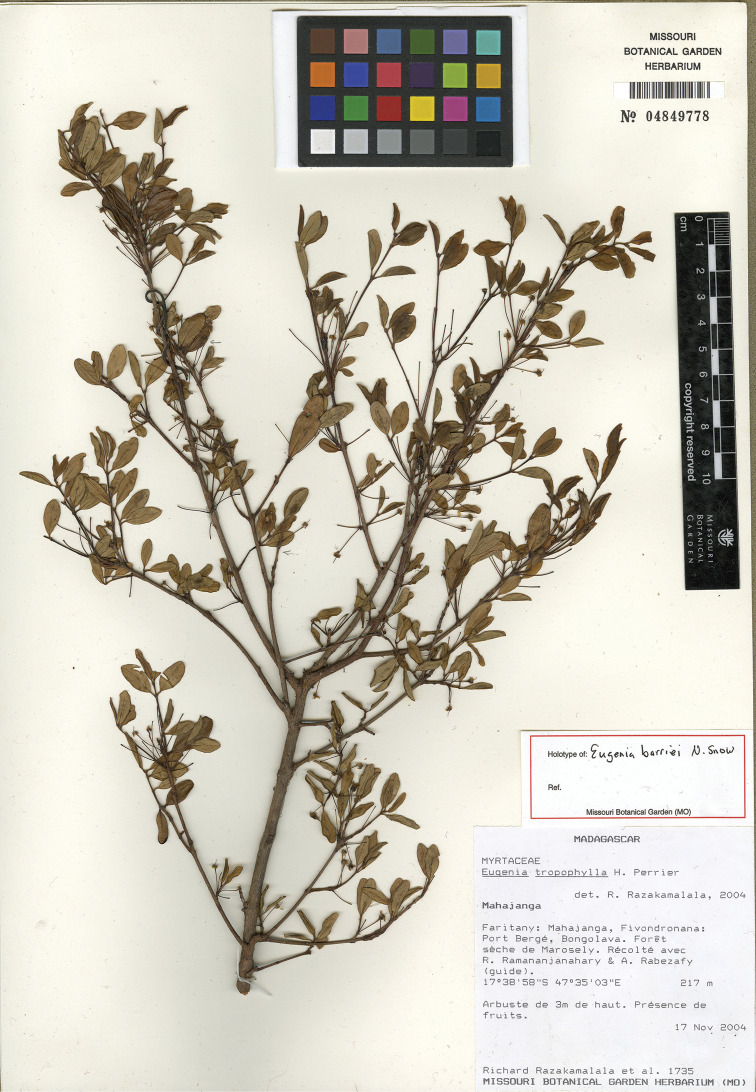
Holotype specimen of *Eugenia barriei* (MO).

N. Snow sp. nov.

urn:lsid:ipni.org:names:77146887-1

holotype: (Figure 3): http://www.tropicos.org/Image/100314913

Haec species a congeneris madagascariensibus pedicellis gracilibus delicatis, foliis tenuiter coriaceis, floribus minutis atque hypanthio dense villoso distinguitur.

#### Type.

Madagascar. Prov. Mahajanga: Fiv. Port Bergé, Marosely, Bongolava, 15°38'58"S, 47°35'03"E, 217 m, 17 Nov. 2004, R. Razakamalala 1735 + R. Ramananjanahary & A. Rabezafy (holotype: MO-4849778!).

#### Description.

Shrubs to 3 m tall; bark of main bole unknown. Vegetative and reproductive parts (where indicated) bearing a moderately dense, shortish indumentum, the individual trichomes dibrachiate or not, appressed to somewhat reflexed (appearing villous), frequently irregularly contorted, whitish or reddish. Branchlets laterally compressed but becoming rounded, smooth, moderately short villous (hairs mostly reflexed and not dibrachiate) becoming glabrous, oil glands common and prominent (after indumentum falls away). Leaves opposite, mostly occurring in 2–4 pairs along seasonal growth of branchlet, thinly coriaceous, venation brochidodromous (invisible to obscure), discolorous, somewhat glossy above but matte below. Axillary colleters absent. Petioles 1.7–2.5 mm long, broadly sulcate above, moderately hairy towards base adaxially in sulcus. Leaf blades 0.9–2.0 (–2.7) × 0.6–0.9 cm, narrowly elliptic or elliptic to narrowly obovate, base cuneate, surface flat to slightly and irregularly (but broadly) sinuous on drying, margin flat or drying slightly revolute, apex obtuse; abaxial surface glabrescent, oil glands common (use magnification) and drying brownish and slightly sunken, midvein flush and becoming imperceptible towards apex; abaxial surface sparsely glabrescent, oil glands relatively sparse to moderate and somewhat less prominent than adaxially, secondary veins few and barely perceptible, the secondaries connected at their ends by a slightly arching pseudo-intramarginal vein 0.3–0.8 mm from leaf margin (i.e., lacking an intramarginal vein distinct from pseudo-intramarginal vein). Inflorescence a monad; the base of the flowering branchlets each with (2–)4–6 flowers arising alternately, each flower subtended by a short, hairy and somewhat ovate to broadly triangular caducous bract. Pedicels (5–)10–15(–20) mm long, 0.3–0.5 mm wide, round in transverse section, stiff, ascending, sparsely hairy (especially near base) to nearly glabrous, moderately glandular throughout, anthopodium present or absent. Bracteoles 2, linear, 1.0–1.2 × 0.3–0.5 mm, sparsely hairy. Hypanthium cupulate 2.0–2.5 mm long, 1.4–1.8 mm wide at base of calyx lobes, densely short-hairy, oil glands absent or sparse (and obscured by hairs); ovary apex glabrous. Calyx lobes 4, 1.5–1.9 mm, broadly ovate to rounded, glabrous on both faces apart from sparse apical hairs (white or reddish), strongly reflexed in athesis. Petals 4 (material scant), ca. 2.5 mm × 2 mm, obovate to widely obovate, glabrous on both faces apart from sparse apical hairs (contorted irregularly), oil glands absent. Staminal region (i.e., lacking a well-defined staminal ring) 1.6–1.8 mm diameter in anthesis, sparsely hairy (trichomes simple); stamens 35–45; filaments 1.5–2.5 mm; anther sacs 0.5–0.7 mm long, globose, basifixed, eglandular. Style 2.5–2.8 mm, glabrous or sparsely hairy basally; stigma narrow and only slightly capitate. Fruit unknown.

#### Etymology.

The specific epithet honors Dr. Fred Barrie (b. 1948) of the Missouri Botanical Garden in recognition of his contributions to our knowledge of *Eugenia* and other genera of Mesoamerican Myrtaceae (e.g., Barrie 2004, 2005).

#### Phenology.

Flowering confirmed only for the middle of November; fruiting likely late November through December.

#### Distribution.

**Figure 4.**
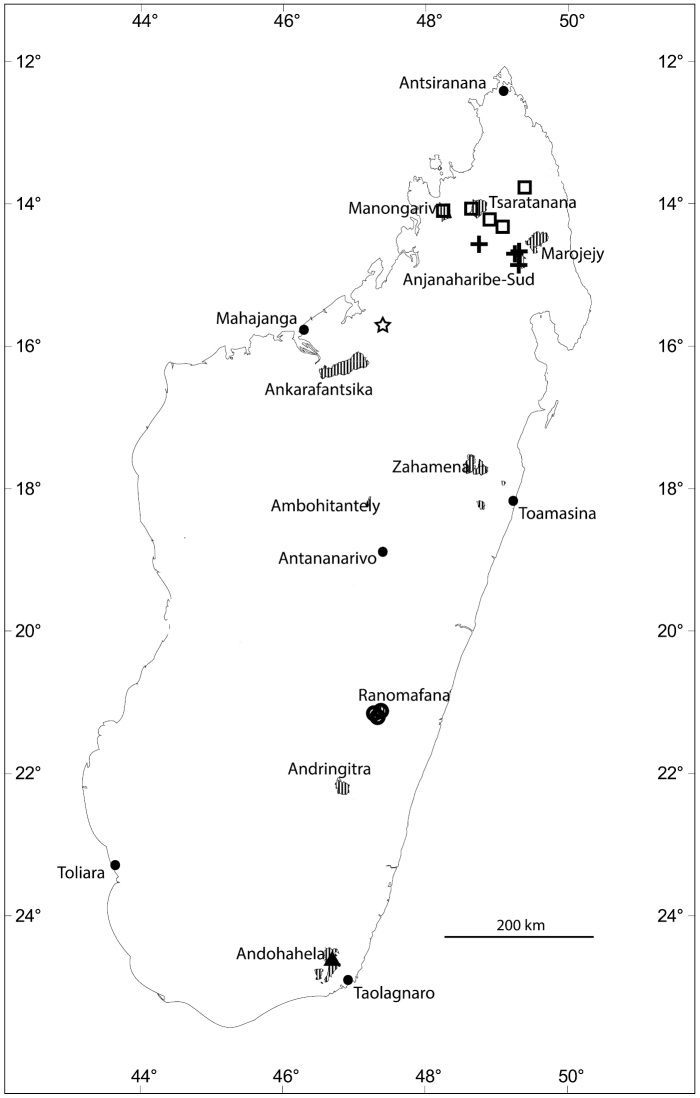
Distribution of new *Eugenia* species in Madagascar with selected Protected Areas (hatched): *Eugenia barriei* (star), *Eugenia delicatissima* (squares), *Eugenia malcomberi* (triangle), *Eugenia ranomafana* (circles), and *Eugenia ravelonarivoi* (crosses).

Known only from near Port Bergé in Mahajanga Province (Figure 4).

#### Conservation status.

With only one collection known from Central-western Madagascar collected in an unprotected and threathened dry forest, *Eugenia barriei* is assigned a preliminary risk of extinction of “Critically Endangered” [CR A3c] following the IUCN Red List Categories and Criteria (IUCN 2012). In the absence of effective protection and the high human pressure on these forests, it is unlikely that the forest will persist beyond 3 generations of *Eugenia barriei* (ca. 30 years).

#### Comments.

The type specimen of *Eugenia barriei* initially was determined as *Eugenia tropophylla* H. Perrier. The latter species and the varieties described by Perrier de la Bâthie (1953a,b) do not represent a single taxon, which even a cursory glance at the numerous syntypes (at P) will reveal.

Among taxa from southeastern Africa, *Eugenia barriei* resembles some specimens of Eugenia capensis subsp. gracilipes. In particular, the slender pedicels of a specimen from Malawi (Chapman 6570 [MO]) have a similar but less dense indumentum on the branchlets, pedicels and hypanthium. Other differences of the Chapman specimen include longer and more densely and prominently punctate leaves, and inflorescences that mostly arise from ramiflorous brachyblasts.

*Eugenia barriei* also has gross morphological similarity to the widespread and relatively common west African species *Eugenia leonensis* Engler & Brehmer (e.g., D.K. Harder 3372 et al. [MO] from Ghana), but it differs from that species by the generally glabrous aspect of the latter. Likewise, *Eugenia barriei* somewhat resembles *Eugenia mufindiensis* Verdc. by virtue of the indumentum of the branchlets, but the latter differs by its much more densely punctate leaves (above and below), the glabrous hypanthium, and a narrower and more deeply sulcate petiole (e.g., M.A. Mwangoka 5945 + H. Mgalla [MO]).

### Eugenia bemangidiensis

Taxon classificationPlantaeMyrtalesMyrtaceae

N. Snow sp. nov.

urn:lsid:ipni.org:names:77146888-1

**Figure 5.**
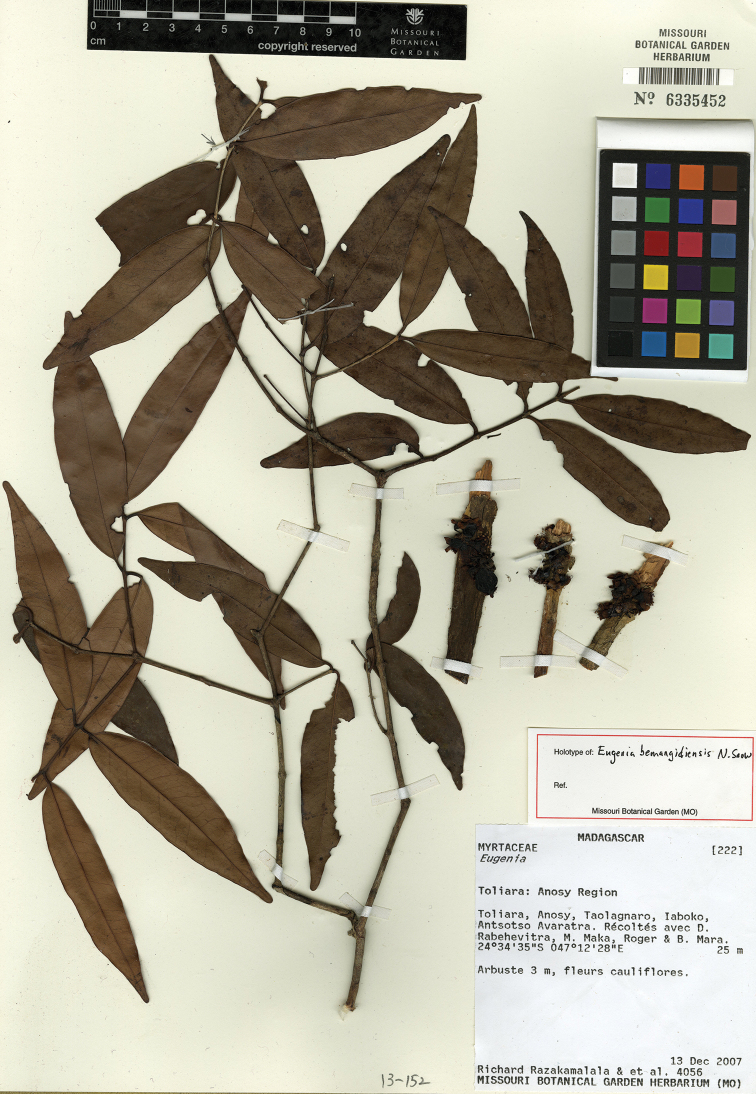
Holotype specimen of *Eugenia bemangidiensis* (MO).

Holotype: (Figure 5): http://www.tropicos.org/Image/100314916

*Haec species* Eugeniae wilsonianae N. Snow *similis, sed ab ea inflorescentiis fasciculatis caulifloris distinguitur; etiam ad altitudines inferiores crescit.*

#### Type.

MADAGASCAR. Prov. Toliara: Anosy, Taolagnaro, Iaboko, Antsotso Avaratra, 24°34'35"S, 47°12'28"E, 25 m, 13 Dec. 2007, R. Razakamalala 4056 + D. Rabehevitra, M. Maka, Roger & B. Mara (holotype: MO-6335452!; isotypes: P [P06490265]!, TAN).

#### Description.

Shrubs 3–4 m; bark of main bole more or less smooth but somewhat flaking, brown to gray. Foliage and reproductive parts glabrous except where noted. Branchlets terete, light brown (dried), smooth, oil glands sparse to moderate (but indistinct). Leaves mostly concentrated near branch tips, coriaceous, slightly discolorus, surfaces matte. Axillary colleters lacking. Petioles 3–4(–5) mm, slightly sulcate above, elgandular. Leaf blades 6–10 × 1.7–3.5 cm, narrowly elliptic to narrowly ovate, base rounded, apex acute to acuminate or a few falcate, margin flat, oil glands not seen (dried material); adaxial midvein broadly but shallowly sulcate, secondary veins indistinct to prominent (with magnification), intramarginal vein 0.5–1.8 mm from edge at midpoint of leaf blade. Inflorescences cauliflorous, arising from amorphous knobby protrusions (short shoots?) at or near nodes; flowers triads, monads, or fascicled (and possibly a few short botryoids); pedicels 1.5–6 mm, flexuous. Bracteoles 2 or absent, ca. 0.5 × 0.5 mm, broadly triangular to broadly rounded, ascending to divergent, stiff. Hypanthium 2.5–4.0 mm, cupuliform, oil glands common but small. Sepals 4, 2–3.0 × 3.5–4 mm, semi-circular to broadly elliptic or oblate, apex broadly rounded to obtuse, lobes very sparsely and minutely ciliate distally, moderately glandular; persistent and crowning (at least) young fruits. Petals 4 (material limited), 5–6 × 3–4 mm, widely elliptic to obovate, glabrous to sparsely and minute ciliate distally, glands sparse to moderate but faint (in dried material). Stamens 50–75, multiseriate; staminal disk short-hairy; ovary apex glabrous but distinctly punctate; filaments 2–5 mm; anthers 0.5–0.8 mm, elliptic, sub-basifixed, connective apex eglandular. Styles 6–7 mm; stigma narrow (scarcely if at all capitate). Fruits ca. 10–15 × 10–15 mm, (material possibly not fully mature) subglobose to globose, base sometimes sharply tapered, glabrous, dark bluish-black (dried).

#### Etymology.

The epithet is derived from the place name *Bemangidy*, a biologically rich region within the provisional Protected Area of Tsitongambarika.

#### Phenology.

Flowering commencing November and December; fruiting likely commencing by late December or early January (unconfirmed).

#### Distribution.

Known only from southeast Madagascar in Toliara Province, ca. 55 km northeast of Taolognaro, in Tsitongambarika (Fig. 2).

#### Habitat and ecology.

Label information is sparse, but based on recent satellite imagery, evidently occurring in moderately to highly disturbed hilly areas that retain thin remnants of forest in ssome drainages and on steeper slopes, the type gathering located some 0.5 km east of much less disturbed primary forests that occur on steeper slopes, the paratype gathering in highly disturbed sites; elevation ca. 25–110 m.

#### Conservation status.

With only three collections known, an AOO of 9 km^2^ and one subpopulation, which is situated outside the current protected area network, *Eugenia bemangidiensis* is assigned a preliminary risk of extinction of “Critically Endangered” [CR A3c+B1ab(iii)] following the IUCN Red List Categories and Criteria (IUCN 2012). All the known material of the species was collected in the proposed new protected area of Tsitonggambarika in the south-east (Fig. 2). The new species seems to have a highly restricted distribution and the lowland evergreen tropical forests where the species grows is under threat. Its definitive protection would likely allow this species to be downlisted to “Endangered”.

#### Comments.

The leaf morphology of *Eugenia bemangidiensis* resembles *Eugenia wilsoniana* (see discussion above). However, the short-pedicellate, fasciculate, and cauliflorous infloresences of *Eugenia bemangidiensis* differ from the axillary, long-pedicellate flowers of *Eugenia wilsoniana*. The type gathering at ca. 25 m elevation of *Eugenia bemangidiensis* is approximately 645 km south of the most southerly collections of *Eugenia wilsoniana*, the latter of which occur at middle elevations of ca. 980–1100 meters.

An indetermined fruiting specimen (Rakotovao 241 [MO-6437191]) from Near Ivohibe approximately 260 km north of the type gathering of *Eugenia bemangidiensis*, which appears to have ramiflorous (and possibly cauliflorous) inflorescences, may be related, although it occurs at 1210 m, far above the known occurrences of *Eugenia bemangidiensis*.

#### Specimens examined.

MADAGASCAR. Prov. Toliara: Iaboko, Antsotso, forêt Ivohibe, 24°33'52"S, 47°14'5"E, 26 Nov. 2005, R. Razakamalala 2326 + E. Ramisa & B. Mara (MO-6433204, P [P06490266]); *ibid. loc.*, 24°34'16"S, 47°12'06"E, 8 Dec. 2007, R. Razakamalala 3799 + D. Rabehevitra, M. Maka, Roger & B. Mara (MO-6308460, P [P04827649]).

### Eugenia calciscopulorum

Taxon classificationPlantaeMyrtalesMyrtaceae

**Figure 6.**
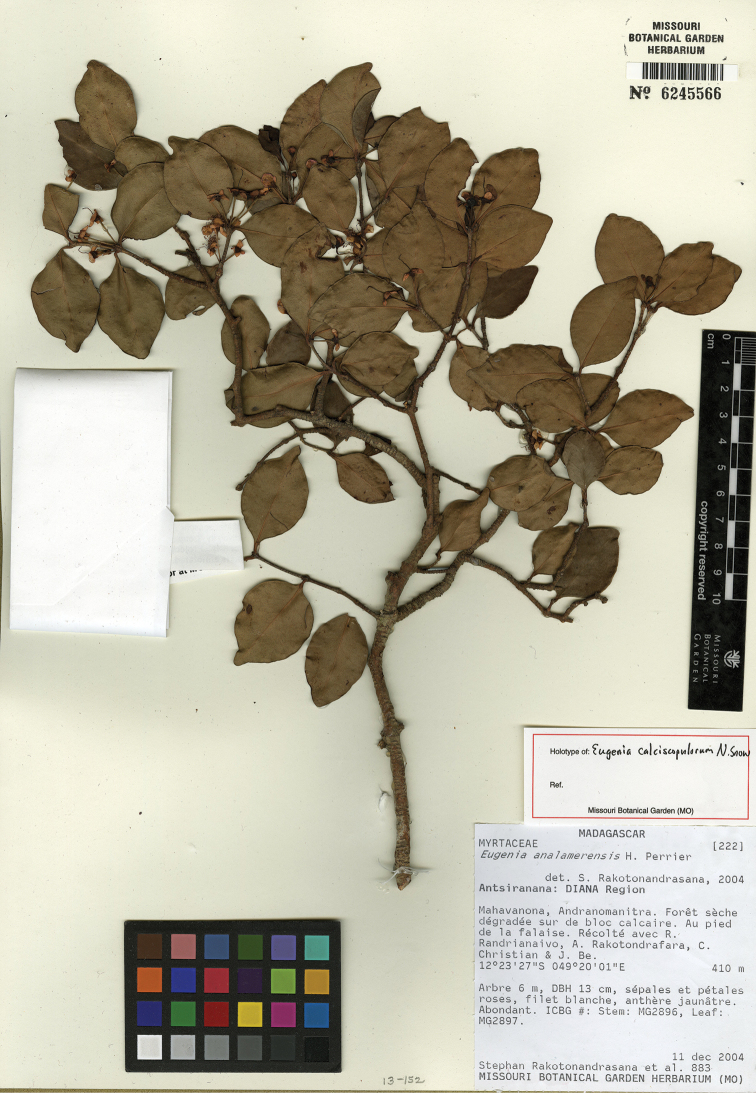
Holotype specimen of *Eugenia calciscopulorum* (MO).

N. Snow sp. nov.

urn:lsid:ipni.org:names:77146889-1

holotype (Figure 6): http://www.tropicos.org/Image/100314917; living material: http://www.tropicos.org/Image/100128169; http://www.tropicos.org/Image/100128168

*Haec species quoad faciem habitumque* Eugeniae richardii (Blume) N. Snow *et al. et* E. hazonjia N. Snow *subsimilis, sed ab eis lamina foliari grosse sinuata, lobis calycinis roseoalbidis atque petalis subroseis distinguitur.*

#### Type.

MADAGASCAR. Prov. Antsiranana: Mahavanona, Andranomanitra, 12°23'27"S, 49°20'01"E, 11 Dec. 2004, S. Rakotonandrasana 883 + R. Randrianaivo, A. Rakotondrafara, C. Christian & J. Be (holotype: MO-6245566!; isotypes: CNARP, KSP [KSP000016]!, P [P04885354]!, TAN).

#### Description.

Trees to 6 m; dbh 13 cm; bark of main bole unknown. Herbage glabrous except as noted. Branchlets rounded; oil glands sparse to common but indistinct; first epidermal layer soon flaking irregularly and drying grayish; later-forming bark smooth, light brown or light gray, frequently cracking horizontally. Leaves coriaceous, mostly concentrated near branch tips; opposite to disjunct opposite, discolorous, surfaces matte. Foliar colleters absent. Petioles 3–4 mm, terete or somewhat sulcate proximally when young, broadening and flattening somewhat distally at base of blade. Leaf blades (1.7–)2.0–4.3 × 1.8–3.0 cm, broadly elliptic (mostly) or a few ovate, base rounded to slightly cuneate, apex obtuse to somewhat acute, margins more or less flat, surface overall broadly sinuous; adaxial midvein sulcate lower 1/3–1/2 becoming flush distally, oil glands common to dense but becoming faint with maturity; abaxial surface oil glands sparse to common but faint at maturity and relatively small, secondary veins relatively indistinct, intramarginal vein 1–2 mm from margin of leaf at midpoint of blade, faint. Inflorescence terminal or axillary, of 1–3 monads per leaf axil. Pedicels 7–15 mm, relatively stiff. Bracteoles 2; ca. 0.5–1.0 mm, ovate, stiff, sparsely short-pubescent distally (trichomes ca. 0.1 mm long). Hypanthium 1.5–2.0 mm, cupuliform to obconic, densely glandular. Calyx lobes 4, 2–3 mm, elliptic to oblong, apex rounded, whitish to pink when fresh, oil glands common and prominently. Petals 4 (material scant), 6–11 × 5–7 mm, elliptic to widely elliptic, sparsely short-ciliate apically, pinkish, oil glands common. Stamens ca. 70–90, multiseriate; filaments 5–8 mm, whitish; anthers 0.6–0.8 mm, globose to elliptic, sub-basifixed, yellowish, the connective bearing a single apical oil gland; staminal disk squarrish, ca. 3 mm across, sparsely short-hairy. Styles 7–9 mm; stigma narrow. Fruits unknown.

#### Etymology.

*Calci* and *scopulorum* combine to form a genitive plural masculine noun, in reference to the locality at the base of a calcareous (limestone) cliff.

#### Phenology.

Flowering December; fruiting most likely late December through January (unconfirmed).

#### Distribution.

**Figure 7.**
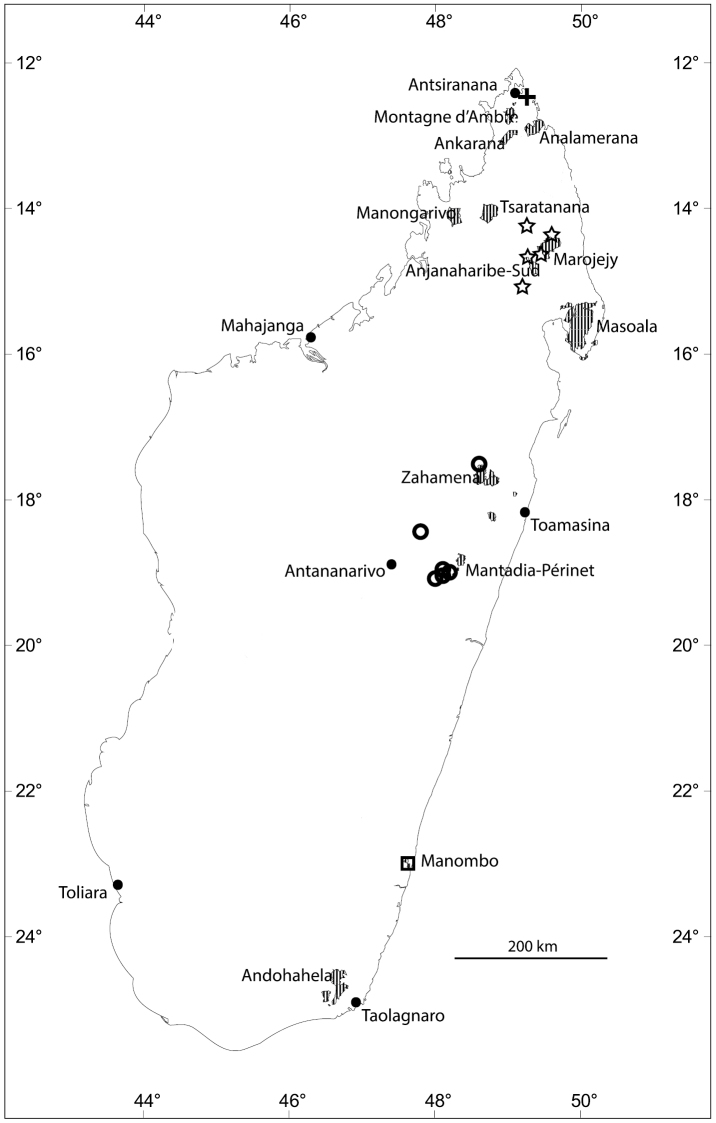
Distribution of new *Eugenia* species in Madagascar with selected Protected Areas (hatched): *Eugenia andapae* (stars), *Eugenia calciscopulorum* (cross), *Eugenia echinulata* (circles), and *Eugenia manomboensis* (square).

Known only from the type gathering in north-eastern Madagascar in Antsiranana, in the Montagne des Français limestone massif (Figure 7).

#### Habitat and ecology.

Degraded dry forest at the foot of a cliff over calcareous substrates; ca. 410 m.

#### Conservation status.

The species was said to be abundant at the type gathering. However, with only one collection known, an AOO of 9 km^2^ and one subpopulation, which is situated within a proposed protected area that currently holds a temporary protection status (Montagne des Français), *Eugenia calciscopulorum* is assigned a preliminary risk of extinction of “Critically Endangered” [CR A3c+B1ab(iii)] following the IUCN Red List Categories and Criteria (IUCN 2012). The species seems to have a highly restricted distribution and the dry deciduous forests around Montagne des Français is mostly degraded as a result of human activities and is under serious threat. Definitive protection of those dry forests likely would allow the species to be downlisted to “Vulnerable”.

#### Comments.

See *Eugenia richardii* for similar species.

### Eugenia delicatissima

Taxon classificationPlantaeMyrtalesMyrtaceae

**Figure 8.**
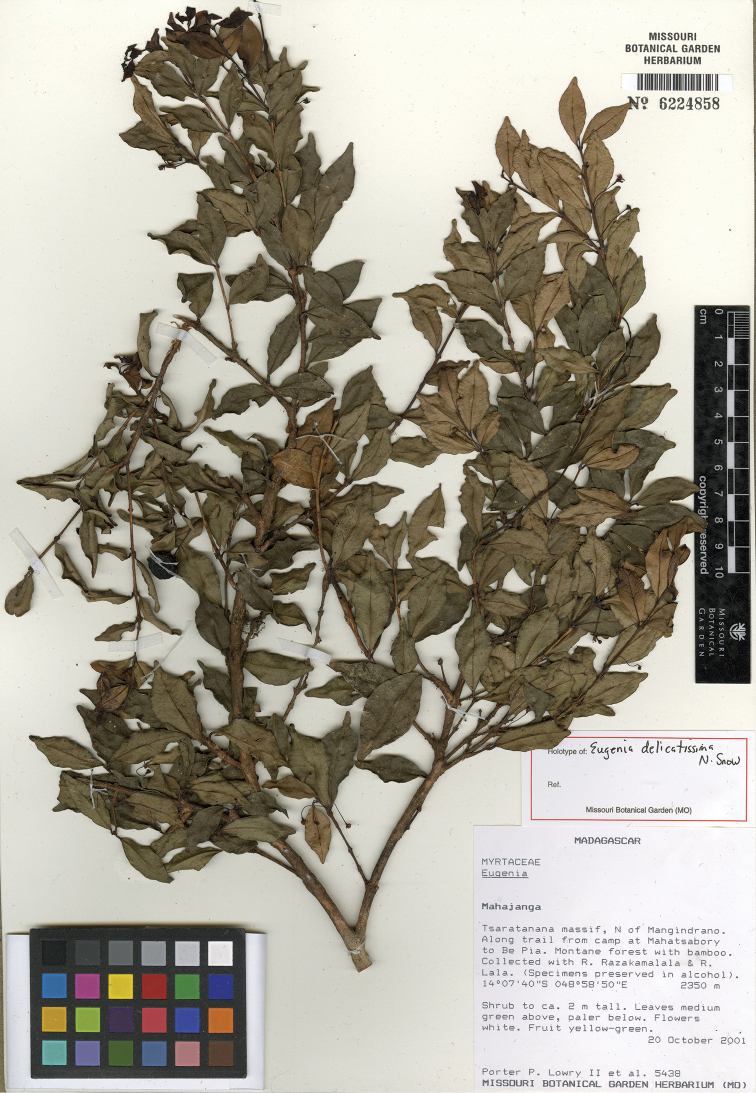
Holotype specimen of *Eugenia delicatissima* (MO).

N. Snow, Callm. & Phillipson sp. nov.

urn:lsid:ipni.org:names:77146890-1

holotype (Figure 8): http://www.tropicos.org/Image/100314909

Haec species a congeneris madagascariensibus lamina foliari tenuiter coriacea irregulariter sinuosa atque pedicello gracili delicato distinguitur.

#### Type.

MADAGASCAR. Prov. Mahajanga: Tsaratanana massif, N of Mangindrano, along trail from camp at Mahatsabory to Be Pia, 14°07'40"S, 48°58'50"E, 2350 m, 20 Oct. 2001, P.P. Lowry II 5438 + R. Razakamalala & R. Lala (holotype: MO-6224858!; isotypes:P [P05208430]!, TAN).

#### Description.

Shrubs to treelets 2–4 m tall; bark of main bole smooth, light brownish or grayish. Plants glabrous or (where noted) sericeous (hairs dibrachiate, reddish). Branchlets laterally compressed, sparsely hairy upon emergence but becoming glabrous, oil glands faint, sparse or occasional and of uniform size, flush or only slightly protruding, smooth, light brown or gray. Leaves evenly distributed along branchlets, thinly coriaceous, venation brochidodromous, strongly discolorous, matte above and below. Axillary colleters if present then soon deciduous. Petioles 4.0–6.5 mm long, slightly to deeply sulcate above, sparsely glabrescent, eglandular. Leaf blades (18–)30–52 mm × (8–)18–25 mm, narrowly elliptic to elliptic (or occasionally broadly elliptic), base cuneate to strongly cuneate, margin flat to slightly revolute (dry), surface slightly irregularly wavy (sometimes including along midvein), apex acute to acuminate, tip obtuse to mostly acute or acuminate or slightly falcate; adaxial surface glabrous, oil glands faint, dense and of uniform size, midvein sulcate but becoming flush distally; abaxial surface glabrous, oil glands faint, sparse to occasional and of different sizes, vein connecting tips of secondaries near margin slightly arching arching only slightly, 0.5–0.9 mm from margin at midpoint of leaf blade. Inflorescence terminal, axillary or ramiflorous, consisting of short brachyblasts with 1–3 pairs of monads, often 2 or more brachyblasts per axil or point of insertion (on naked branches), or monads arising in axils. Pedicels 3–11 mm long, 0.3–0.4 mm thick, stiff to somewhat flexuous, ascending to sometimes reflexed, glabrous or very sparsely short-hairy, lightly striate, round, oil glands sparse and faint, anthopodia and metaxyphylls absent. Bracteoles 0.8–1.3 mm long, less than 0.5 broad (at base), very narrowly triangular to ovate, normally persisting in anthesis, sparsely hairy (or merely ciliate). Hypanthium obconic, (0.8–)1.2–1.3 mm long, 0.9–1.0 mm broad at apex beneath base of calyx lobes, sparsely hairy to glabrous, smooth, oil glands sparse to common and protruding. Calyx lobes 4, 1.3–1.9 mm long, broadly ovate to rounded, sparsely ciliate, sparsely glandular, greenish. Petals 4, 2.8–4.4 × 1.6–1.8 mm, obovate to broadly elliptic, glabrous or sparsely ciliate apically, white, oil glands sparse to common, of uniform size and somewhat protruding below. Staminal disk ca. 1.8 mm in diameter, sparsely hairy. Stamens 15–25; filaments 3–4 mm long; anther sacs ca. 0.5 mm long, globose to subcylindrical, dorsifixed, brown, connective with an apical gland. Style 4–5 mm long, glabrous; stigma narrow. Fruit 0.9–1.4 × 0.8–1.6 cm, subglobose to globose, texture smooth, rounded or tapered at base, glabrous, greenish but drying blackish, calyx lobes reflexed flat against apex.

#### Etymology.

A superlative of *delicata* in Latin, in reference to the thin, almost thread-like pedicels.

#### Vernancular name.

Rotramadinika (Rakoto 294).

#### Phenology.

Flowering in late October through at least early November; fruiting November.

#### Distribution.

Known from the northern mountains of Madagascar in and around the Manongarivo and Tsaratanana protected areas and in the Sorata region (Fig. 4).

#### Habitat and ecology.

High elevation montane forests with bamboo; 1100–2350 m.

#### Conservation status.

With an EOO of 3278 km^2^, an AOO of 36 km^2^ and four subpopulations, two of which are situated within the protected area network (Manongarivo, Tsaratanana), *Eugenia delicatissima* is assigned a preliminary risk of extinction of “Vulnerable” [VU B1ab(iii)+2ab(iii)] following the IUCN Red List Categories and Criteria (IUCN 2012). At the summit of Beampoko *Eugenia delicatissima* was indicated as being rare (Rakotovao 2565).

#### Comments.

*Eugenia delicatissima* is part of an apparent species group characterized by thinly coriaceous, sinuous leaf blades and slender, often elongate and delicate pedicels.

The thinly coriaceous, sinuous leaf blades of *Eugenia delicatissima* are somewhat suggestive of *Eugenia echinulata*, but the fruits of *Eugenia delicatissima* are smooth (e.g. Randriambololomamonjy 270), unlike the highly irregular (warty to echinate) texture of of *Eugenia echinulata*.

The slender pedicels are similar to those of *Eugenia tropophylla* H. Perrier, a highly heterogenous species that Perrier de la Bâthie (1953a: 167) initially described with three subspecies, none of which match *Eugenia delicatissima*. The taxonomy of *Eugenia tropophylla* needs serious revision and will be the subject of a forthcoming publication.

An indetermined specimen of *Eugenia* from Mayotte in the Comoros (Barthelat 992 [G, K, MO]) also has slender pedicles, but they are much shorter than those of *Eugenia delicatissima*. The leaf blades of the Barthelat specimen are longer and the bases rounded, unlike the cuneate bases of *Eugenia delicatissima*. Taxa from eastern and southern Africa with delicate pedicels include Eugenia capensis (Eckl. & Zeyh.) Sonder subsp. gracilipes F. White (e.g., P. van Wyk BSA 2844 [MO] from Zimbabwe and J.D. Chapman 6570 [MO] from Malawi). Another species from Africa with delicate pedicels that differs from *Eugenia delicatissima* in various traits is *Eugenia congolensis* De Willd & T. Durand (e.g., J. Madidi 642 et al. [MO] and F. Bujo 602 [MO] from Congo). None of these, however, are good morphological matches for *Eugenia delicatissima*.

#### Specimens examined.

MADAGASCAR. Prov. Antsiranana: Massif du Manongarivo, [13°59'24"S, 48°22'12"E], c. 1600 m, 1909, Perrier de la Bâthie 6503 (P [P05258587]); Sommet de Beampoko, Ambohimirahavavy, 14°13'55"S, 49°08'23"E, 2137 m, 21 Nov. 2005, C. Rakotovao 2565 (G, MO-6174672); Vohemar, Andrafainkona, Ampsarahina, forêt de Maromaniry située à 5 km au N d’Ampiarahina, 13°38'49"S, 49°32'13"E, 1177 m, 7 Nov. 2007, O. Randriambololomamonjy 270 + R. Razakamalala & Jaowind (MO-6186659); Massif du Tsaratanana, crête (et ses abords) séparant les bassins du Sambirano et de la Mahavavy, entre l’Andohanisamborano et la cote 2362, 9-10 Nov. 1966, Service Forestier 27019 (P [P05097488]).

### Eugenia echinulata

Taxon classificationPlantaeMyrtalesMyrtaceae

**Figure 9.**
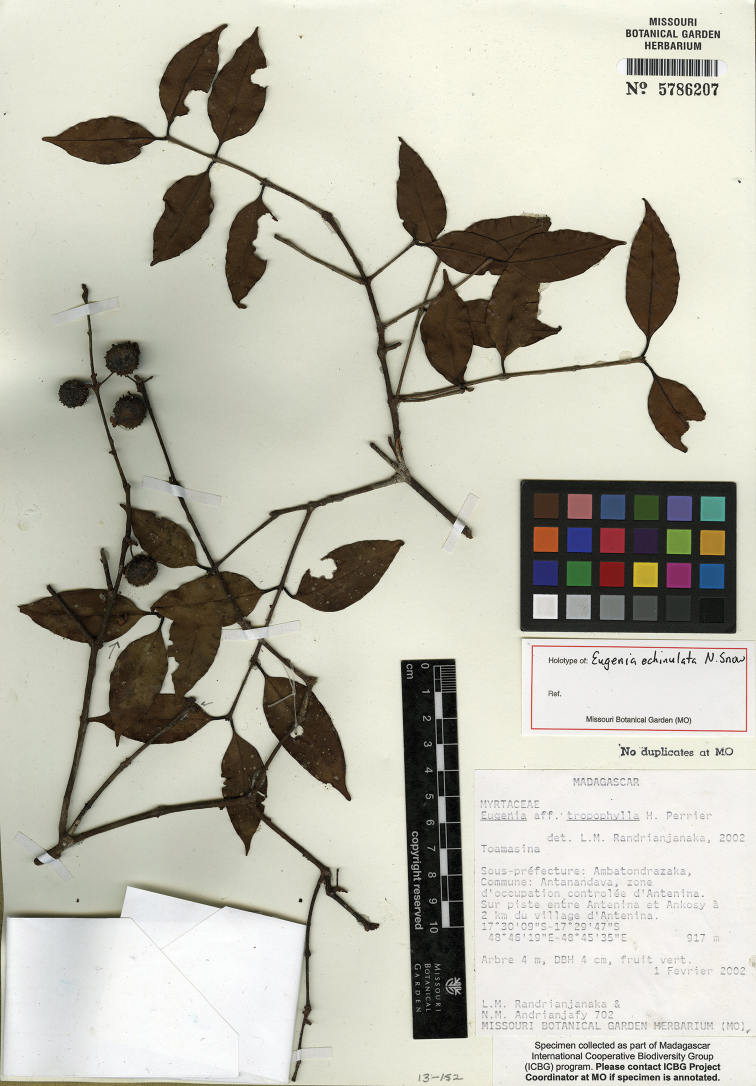
Holotype specimen of *Eugenia echinulata* (MO).

N. Snow sp. nov.

urn:lsid:ipni.org:names:77146891-1

holotype (Figure 9): http://www.tropicos.org/Image/100314922; living material: http://www.tropicos.org/Image/100306108

Haec species inter congeneros madagascarienses fructibus maturis valde echinulatis distinguitur.

#### Type.

Madagascar. Prov. Toamasina: zone d’occupation controlée d’Antenina, sur piste entre Antenina et Ankosy, à 2 km du village d’Antenina, 17°30'00’’–17°29'47"S, 48°46'19"E–48°45'35"E, 917 m, 1 Feb. 2002, L.M. Randrianjanaka 702 + N.M. Andrianjafy (holotype: MO-5786207!; isotypes: P, TAN).

#### Description.

Shrubs or trees 1.5–4 m tall; dbh 4–5 cm; bark of main bole brown, somewhat cracking. Foliage and flowers glabrous except as noted. Branchlets terete to laterally compressed, drying light brown to gray, smooth, oil glands occasional but barely visible. Leaves slightly discolorous, more or less evenly distributed, margins coarsely sinuous, venation brochidodromous, surfaces matte. Axillary colleters absent. Petioles 4–6 mm long, slightly suclate, eglandular. Leaf blades (3.0–)3.8–6.5 × (1.0–)1.6–3.5 cm, narrowly elliptic or narrowly ovate to elliptic, base cuneate, apex acute to acuminate; adaxial midvein sulcate proximally but becoming flush distally, oil glands dense but faint, sometimes protruding slightly on dried material; abaxial surface midvein prominently raised, oil glands common to dense and of uniform size but faint, sometimes slightly protruding (dried material). Inflorescence ramiflorous or axillary. Flowers solitary or clustered into short brachyblasts; brachyblasts 2–5 mm long, densely short-bracteolate, sparsely covered with twisted, whitish-maroon trichomes. Pedicles 5–7.5 mm, glabrous. Flower material scant. Hypanthium ca. 3 mm, coarsely large-pustullate (warty), light green or yellowish-green in bud. Calyx lobes 4 (rose-colored in bud), 3–4 mm, elliptic to broadly ovate or rounded, glabrous, mostly persisting and crowning fruit. Petals not seen; said to be pink (Antilahimena 8858). Stamens not seen; anthers said to be yellow with white filaments (Ahtilahimena 8858). Style not seen. Berry 1.3–1.8 × 1.0–1.5 cm, globose to subglobose, coarsely rugose-warty when fresh but drying echinulate (with with short sharp points), densely glandular when younger, green when immature, drying nearly black. Seeds in mature fruit 1; ca. 10–11 mm, globular, outer seed coat elgandular.

#### Etymology.

The specific epithet comes from the Latin *echinulatus* (having very small prickles) in reference to the prickly, dried, mature fruits that are unique for the genus in Madagascar.

#### Vernacular name.

Rotra (Antilahimena 6841).

#### Phenology.

Flowering December; fruiting January and February.

#### Distribution.

Known from the eastern escarpment of Madagascar in the Toamasina Province around Analamazaotra and Ambatovy and in the highlands at Anjozorobe in Antananarivo Province (Fig. 7).

#### Habitat and ecology.

Dense humid evergreen forests; ca. 915–1350 m.

#### Conservation status.

One collection indicated the species as being abundant in its area of collection (Randrianaivo 1463 et al.). However, with an EOO of 4,372 km^2^, an AOO of 72 km^2^ and three subpopulations, two of which are situated within the protected area network (Analamazaotra, Zahamena), *Eugenia echinulata* is assigned a preliminary risk of extinction of “Vulnerable” [VU B1ab(iii)+2ab(iii)] following the IUCN Red List Categories and Criteria (IUCN 2012).

#### Specimens examined.

MADAGASCAR. Prov. Antananarivo: Reg. Analamanga, Anjozorobe, Ambongamarina, Ampamoa, à 10 km au Nord-Est d’Anjozorobe, prolongement N de la fôret d’Antsahabe, 18°23'19"S, 47°55'49"E, 1352 m, 15 Feb. 2007, R. Randrianaivo 1463 + L. Vary, François, Jacques & Rakoto (MO-6440448). Prov. Toamasina: Alaotra-Mangoro, Fkt: Menalamba, Ambatovy, 1168 m, 18°50'11"S, 48°18'44"E, 22 Jan. 2007, P. Antilahimena 5170 et al. (MO-5786207, KSP [KSP000006], P [P00730617]); *ibid. loc.*, 18°52'25"S, 48°20'44"E, 979 m, 4 Oct. 2008, P. Antilahimena 6566 et al. (MO, P, TAN); *ibid. loc*., 18°52'28"S, 48°17'37"E, 1010 m, 10 Nov. 2008, P. Antilahimena 6841 + M. Ratolojanahary, B.A. Ratodimanana, M. Randrianarivony, M. Ratovomanana & F. Edmond (MO, P, TAN); *ibid. loc*., 18°52'30"S, 48°17'35"E, 970 m, 10 Nov. 2008, P. Antilahimena et al. 6854 (MO, P, TAN); *ibid. loc*., 18°55'22"S, 48°25'39"E, 1013 m, 21 Dec. 2013, Antilahimena 8858 + Rabarison, Honoré, Félix, Randrimitantsoa Jean & V. Razafindrahaja (MO, P, TAN); Phelps Dodge project site, ca. 15 air-km NE of Moramanga, ca. 11 km E of Antanambao. Ambatovy, SE valley (Ambohimanga), 18°51'34”S, 48°18'25”E, 1050 m, P.J. Rakotomalaza 1208a (MO, P [P00730620]); Analamazaotra, Ambatovy, 18°56'10”S, 48°25'33”E, 11 Dec. 2013, Ramahenina 276 et al. (MO); Ambatovy, forêt de Savihara, 18°52'06"S, 48°16'43"E, 22 Feb. 2010, D. Razafimelison 10 et al. (P [P00730618]); Alaotra-Mangoro, Moramanga, Andasibe, Réserve Spécial d’Analamazaotra, Circuit Indri 1, 18°56'20"S, 48°25'09"E, 947 m, 14 Dec. 2013, H.M.J. Rasoazanany 541 + H. Razafindraibe, B. Ramandimbisoa & Rakotondravelo (MO, P, TAN).

#### Comments.

*Eugenia echinulata* is unknown in flower apart from one remnant hypanthium on the holotype. However, the coarsely sinuate leaf blades and acuminate apices, coupled with the warty mature fruits, are diagnostic and unknown in combination elsewhere among Malagasy congeners. Whereas *Eugenia muscicola* H. Perrier has narrowly elliptic leaves, its leaf margins are more or less planar. A number of similar sterile collections from this area may be this taxon.

The texture of the fruit is coarsely rugulose-warty (see link above to image of living material), but the warty protrusions collapse to a large extent and upon drying and then assume a sharper, echinulate texture.

### Eugenia gandhii

Taxon classificationPlantaeMyrtalesMyrtaceae

**Figure 10.**
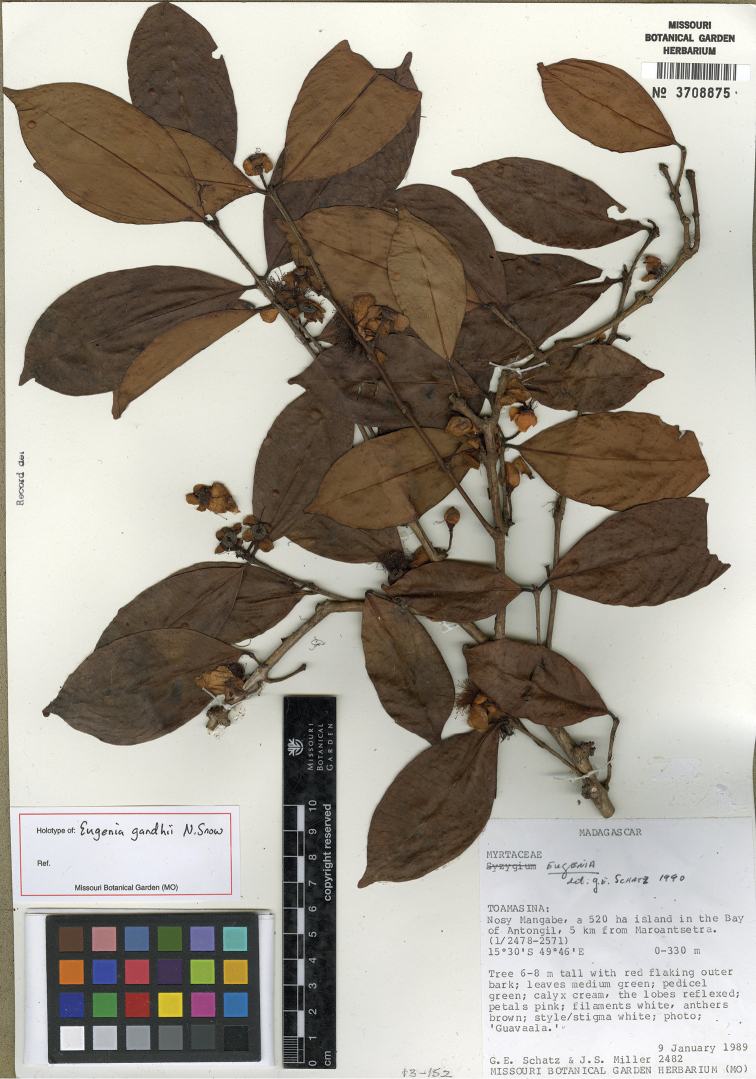
Holotype specimen of *Eugenia gandhii* (MO).

N. Snow sp. nov.

urn:lsid:ipni.org:names:77146892-1

holotype (Figure 10): http://www.tropicos.org/Image/100314906; living material: http://www.tropicos.org/Image/100160915

Haec species a congeneris madagascariensibus lamina foliari tenuiter coriacea elliptica usque obovata dense sed inconspicue glandulosa apice acuta acuminatave distinguitur.

#### Type.

MADAGASCAR. Prov. Toamasina: Nosy Mangabe, a 520 ha island in the Bay of Antongil, 5 km from Maroantsetra, 15°30'S, 49°46'E, 0–330 m, 9 Jan. 1989, G.E. Schatz 2482 + J.S. Miller (holotype: MO-4805069!; isotypes: MO-3708875!, P [P05260197]!, TAN).

#### Synonym.

Eugenia arthroopoda Drake var. ambalavensis H. Perrier in Mém. Inst. Sci. Madagascar, Sér. B, Biol. Vég. 4(2): 177. 1953. Nom. inval. (lacking Latin diagnosis, ICN, Art. 39.1, McNeill et al. 2012).

#### Description.

Shrubs to trees, 3–12 m. Trunk 3–8 cm dbh; outer bark of main bole reddish, papery to flakey and peeling. Foliage and reproductive parts glabrous except where noted. Branchlets terete to laterally compressed, reddish when fresh but drying brownish or grayish, glabrous but prominently glandular, the epidermis smooth but soon peeling (dried specimens) and often thin and narrow grayish strips or even threads. Leaves opposite, medium green (fresh), slightly to strongly discolorous, venation brochidodromous, thinly coriaceous (easily cracking when dried), surfaces matte. Axillary colleters lacking. Petioles 2.8–6 mm, slightly sulcate distally. Leaf blades (5.5–)9–16 × 2.5–6.0 cm, elliptic to occasionally obovate, base cuneate, apex acute to mostly acuminate or caudate, margins flat; adaxial surface glabrous, densely punctate (glands small and best seen with magnification); abaxial surface similar, lateral veins indistinct to prominent; intramarginal vein 1–3.5 mm from margin at midpoint of blade. Inflorescence terminal, axillary, or ramiflorous, of soliltary monads or in fascicles of 2–3 flowers. Pedicels 9–29 mm long × 0.8–1.2 mm thick, stiff, moderately glandular, ascending to erect, green. Bracteoles narrowly ovate to broadly rounded, 0.5–1.3 × 1.0–1.2 mm, stiff but thin, glabrous to minutely and sparsely sericeous on margin, often persistent in fruit. Hypanthium 4–5 mm long, cupuliform, densely glandular (some glands larger proximally). Calyx lobes 4, 6–9 × up to 11 mm, broadly elliptic to rounded, cream-colored (fresh), reflexed in flower, persistent and often crowning the fruit. Petals 4, 11–14 × up to 12 mm, widely elliptic to widely obovate (narrowly greatly at base), sparsely and minute ciliate marginally, densely glandular (glands of variable size but typically large proximally). Stamens 250–300, multiseriate, exserted; staminal disk short-hairy (trichomes slightly ferrugineous); filaments 8–15 mm, white; anthers globose, 0.3–0.5 mm, brownish, bearing a single large apical gland. Ovary apex glabrous but somewhat glandular. Style 8–10 mm; stigma narrow (scarcely if at all swollen). Berries 2.5–4.0 × 3.0–4.0 cm, subglobose to globose, purplish or violet (drying nearly black). Locules 2; placentation axile. Seeds up to 20 mm long and wide (available material possibly not fully mature), up to 6 per fruit, evidently often somewhat flattened, cotyledons and hypocotyl barely if at all differentiated.

#### Etymology.

The species honors Dr. Kanchi N. Gandhi (b. 1948) of Harvard University Herbaria, North American editor of the *International Plants Names Index*, and an expert of botanical nomenclature who has assisted colleagues with the proper usage of botanical Latin and interpretation of the Code over many years.

#### Vernacular names.

Gavoala (Ravelonarivo 103); gavoalabe.

The name “guaavaala” appears on one specimen, but native speakers inform us that it is a nonsensical name based on a combination of the common English name *guava* (for members of *Psidium*, a Neotropical genus) and *ala*, meaning locally “of the forest”.

#### Phenology.

Flowering January through June; fruiting March through November.

#### Distribution.

**Figure 11.**
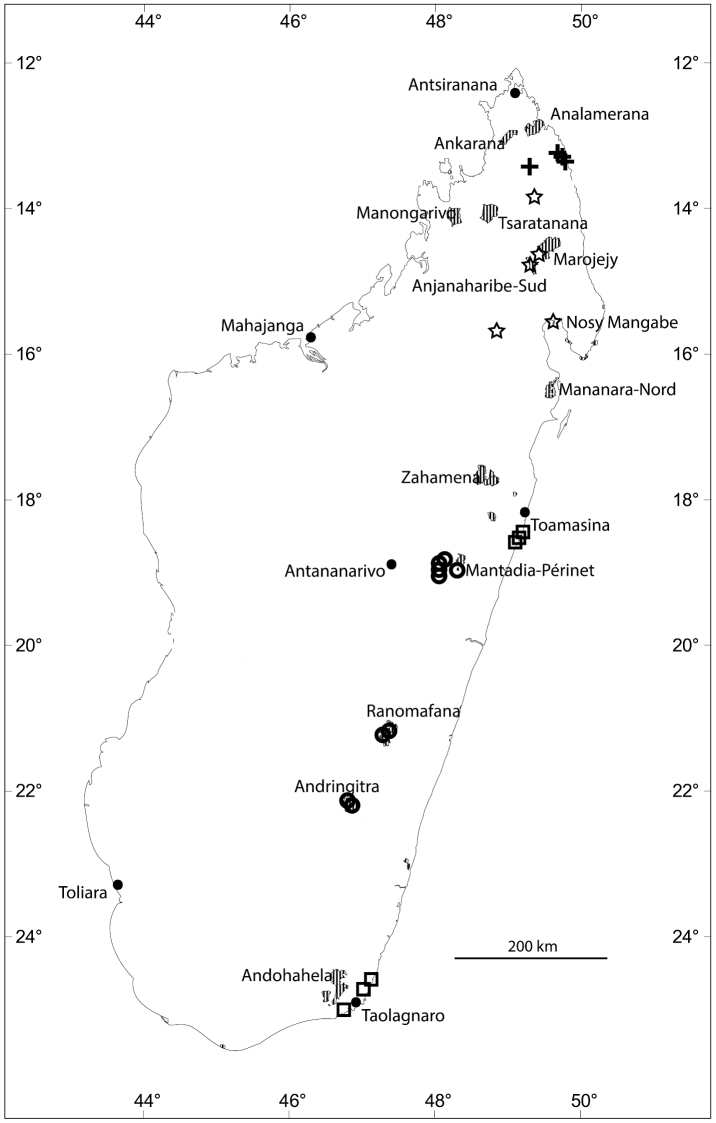
Distribution of new *Eugenia* species in Madagascar with selected Protected Areas (hatched): *Eugenia gandhii* (stars), *Eugenia hazonjia* (crosses), *Eugenia iantarensis* (circles), and *Eugenia obovatifolia* (squares).

Endemic to northeast Madagascar, recorded from the Island of Nosy Mangabe, the Masoala Peninsula, and in and around the Anjaniharibe Reserve (Figure 11).

#### Habitat and ecology.

Humid and typically dense forests; 0–1424 meters.

#### Conservation status.

With an “Extent Of Occurrence” (EOO) of 9,504 km^2^, an “Area Of Occupancy” (AOO) of 45 km^2^ and five subpopulations, three of which are situated within the protected area network (Anjanaharibe-Sud, Marojejy and Nosy Mangabe), *Eugenia gandhii* is assigned a preliminary status of “Least Concern” [LC] following the IUCN Red List Categories and Criteria (IUCN 2012).

#### Comments.

Perrier de la Bâthie’s Eugenia arthroopoda H. Perrier var. ambalavensis H. Perrier was based on a single collection from the lowlands just to the south of Marojejy Reserve, but we have little doubt that it is conspecific with the material we include in *Eugenia gandhii*, which ranges from the nearby Anjaniharibe Reserve to the southwest of Marojejy to the Masoala peninsula and Nosy Mangabe farther south.

The thinly coriaceous leaf blades of *Eugenia gandhii* are suggestive of *Eugenia vatomandrensis* H. Perrier, which thus far is known as a coastal species from near Vatomandry north towards Soanierana (Perrier 1953a). However, the leaves of *Eugenia vatomandrensis* are rounded to subcordate and nearly sessisle, which contrast with the distinctly petiolate, cuneate leaf blades of *Eugenia gandhii*.

#### Specimens examined.

MADAGASCAR. Prov. Antsiranana: Vallee de la Lokoho (Nord-est), près d’Amalavanonio, 9-10 January 1949, H. Humbert + G. Cours 22804 (P [P00118089, P00118090]!); Réserve Spéciale d’Anjanaharibe-Sud, aux environs du sommet, 1161–1424m, 14°46'15"S, 49°28'00"E, 21 Mar.–7 Apr. 1994, D. Ravelonarivo 103 et al. (KSP [KSP000010, KSP000011], MO-6135429, P [P04884879]); Réserve Spéciale Anjanaharibe-Sud, Ambodisatrana, aux environs des sommets, 14°32'45"S, 49°35'15"E, 809–1364 m, 25 May–3 Jun. 1994, D. Ravelonarivo et al. 174 (KSP [KSP000013]); Réserve Spéciale d’Anjanaharibe-Sud, village d’Andranotsarabe, suivant la route Nationale d’Andapa-Bealanana de la piste vers à l’W, Ambatomainty, Camp No. 2, 14°44'42"S, 49°27'42"E, 1185–1335 m, 3 Nov. 1994, D. Ravelonarivo 516 + P. Rabesonina (KSP [KSP000012], P[P04885352]); Réserve Spéciale Anjanaharibe-Sud, suivant la piste pour Ambalaheva, haute rivière d’Andramonta, 14°36'40"S, 49°24'12"E, 628–1879 m, 22 Feb. 1996, D. Ravelonarivo et al. 929 (KSP [KSP000015, KSP000014], MO-6135429, P [P05208458]). Toamasina: Maroantsera Tampolo, Péninsule Masoala, env. 1 km NW du camp (site du posé du radeau), 15°42'77"S, 48°58'25"E, 100–200 m, 1 Nov. 2001, O. Poncy 1548 + S. Rapanarivo (K, P [P00373064], TEF); Nosy Mangabe, 5 km from Maroantsetra in the Bay of Antongil, 15°30'S, 49°46'E: all sterile specimens collected by G.E. Schatz + A. Gentry from 13–23 April 1988: 2010 (MO-3599534), 2020 (MO-3599524), 2100 (MO-3598203), 2137 (MO-3598167; P [P05156045]), 2197 (MO-3596402), 2210 (MO-3596403), 2261 (MO-3597989), 2294 (MO-3597957), 2296 (MO-3597954).

### Eugenia hazonjia

Taxon classificationPlantaeMyrtalesMyrtaceae

**Figure 12.**
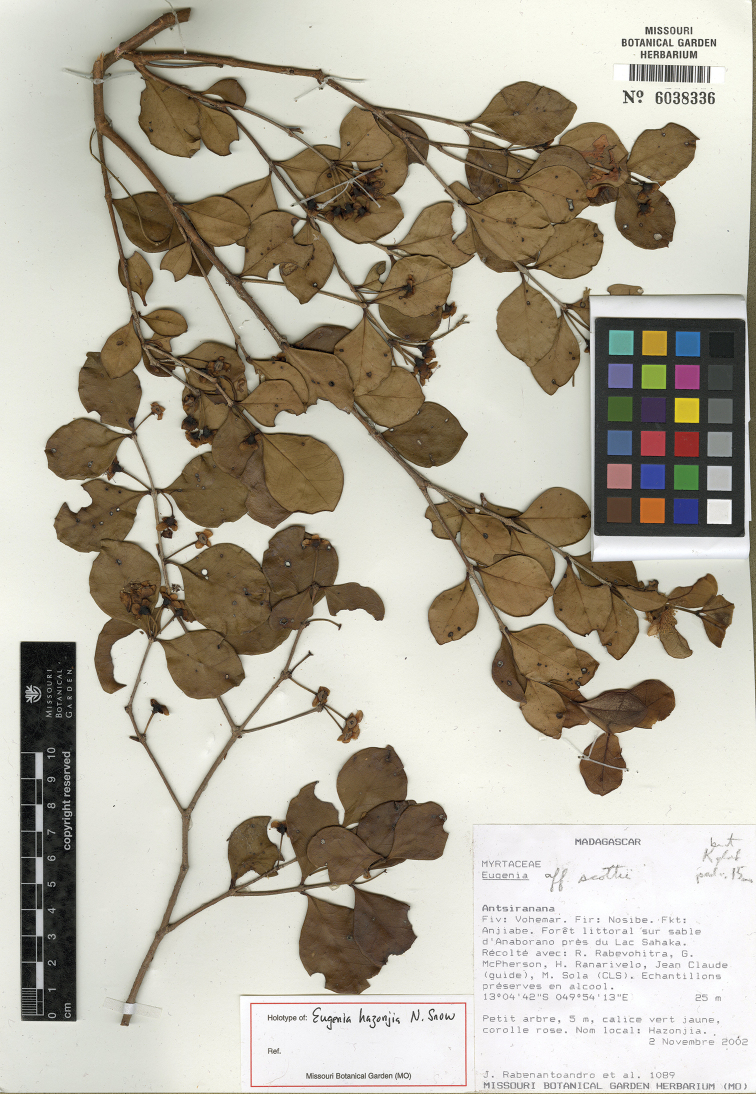
Holotype specimen of *Eugenia hazonjia* (MO).

N. Snow sp. nov.

urn:lsid:ipni.org:names:77146893-1

holotype (Figure 12): http://www.tropicos.org/Image/100314908

Haec species a congeneris madagascariensibus ramificationis ordinatione V-formi, lamina foliari elliptica vel late elliptica basi cuneata apice rotundata usque late acuta, costa adaxialiter plana atque bracteolis cupulatis base latis distinguitur.

#### Type.

MADAGASCAR. Prov. Antsiranana: Fiv. Vohemar. Fir: Nosibe. Fkt: Anjiabe, 13°04'42"S, 49°54'13"E, 25 m, 2 Nov. 2002, J. Rabenantoandro 1089 + R. Rabevohitra, G. McPherson, H. Ranarivelo, C. Claude & M. Sola (holotype: MO-6038336!, isotype: P [P05208261]!).

#### Description.

Shrubs or small trees 2–5 m; dbh 5 cm; bark of main bole unknown. Herbage glabrous except as noted. Branchlets strongly laterally compressed on emergence but becoming terete, drying light brown to light gray, sometimes slightly striate, oil glands sparse. Leaves mostly evenly distributed, coriaceous, venation brochidodromous, discolorous, surfaces matte and sometimes slightly undulate. Axillary colleters absent. Petioles 1–3 mm, flattened adaxially, sometimes slightly sulcate basally. Leaf blades (2.0–)2.5–4.0 × (1.0–)1.5–3.3 cm, elliptic to broadly elliptic, base cuneate, apex rounded or obtuse to (occasionally) broadly acute, margins usually slightly revolute; adaxial surface oil glands common to dense but faint, midvein flush; abaxial surface midvein flush except proximally, oil glands common to dense (sometimes faint), secondary veins indistinct, intramarginal vein 1–2 mm from edge at midpoint of blade. Inflorescence of terminal or axillary monads, these solitary, paired, or (especially at apex) fasciculate. Pedicels 8–18 mm, ascending, stiff. Bracteoles 2, 1.0–1.5 mm, broadly ovate and cuplike (sometimes keeled), rigid, minutely and sparsely ciliate proximally (use magnification). Hypanthium 2.5–3.0 mm, cupuliform, oil glands dense. Calyx lobes 4, 3–5 mm, broadly rounded, apex obtuse, sparsely short ciliate, prominently glandular, greenish. Petals 4, 8–11 × 6–7 mm, obovate to broadly obovate, glabrous, pink. Stamens >150, multiseriate, staminal disk densely short-hairy; filaments 5–9 mm; anthers 0.6–0.9 mm, ellipsoid, basifixed, connectives with a single (but faint) apical gland. Styles up to 8 mm; stigma narrow and scarcely if at all capitate. Fruits unknown.

#### Etymology.

Named after the local vernacular name *hazonjia*, which is recorded on two specimen labels.

#### Phenology.

Flowering September through December; fruiting unknown.

#### Distribution.

Known from Antsiranana Province in north-eastern Madagascar approximately 30 km northwest of Vohemar and in the Daraina region (Fig. 11).

#### Habitat and ecology.

From littoral forest on sand and dry deciduous forest; 15–1015 m.

#### Conservation status.

With an EOO of 39 km^2^, an AOO of 27 km^2^ and two subpopulations, one of which is located within in the temporarily protected Loky-Manambato area, *Eugenia hazonjia* is assigned a preliminary risk of extinction of “Endangered” [EN B1ab(iii)+2ab(iii)] following the IUCN Red List Categories and Criteria (IUCN 2012). The new species appears to be hightly restricted in its distribution, and the lowland evergreen tropical and littoral forests where it grows is under threat. Its definitive protection likely would allow downlisting to “Vulnerable”.

#### Comments.

*Eugenia hazonjia* somewhat resembles *Eugenia urschiana* H. Perrier, but the latter has longer petioles and shorter pedicels. It resembles even more closely a specimen (Rabevohitra 5044 et al.) form the eastern side of Ile Sainte Marie approximately 420 km south, but which has a moderately dense indumentum on the hypanthium and pedicels of dibrachiate, ferrugineous trichomes, and a staminal disk with significantly longer and more erect trichomes.

#### Specimens examined.

MADAGASCAR. Prov. Antsiranana: Vohemar, Nosibe, Fkt: Anjiabe, Anaborano, Fôret littorale d’Analabe, Mosorolava, 13°05'41"S, 49°54'21"E, 24 Sept. 2004, H. Manjakahery 17 (MO-6308382); Vohemar, Nosy-Be, Anjiabe, forêt de Lac Sahaka, Anaborano, 13°04'49"S, 49°54'14"E, 9 Dec. 2007, R. Randrianaivo 1505 + S. Randrianasolo, R. Rakotondrajaona, V. Beninjara, C. Claude, Cyprien & M. Sola (G, MO-6250496); Loky Manambato, Daraina, forêt d’Antsahabe, 13°12'59”S, 49°32'47”E, 1015 m, 1 Dec. 2004, Gautier 4799 (G, P [P05094974]); *ibid. loc.*, 900 m, 6. Dec. 2004, L. Gautier 4842 + L. Nusbaumer (G [G00019226], P [P04827946], TEF n.v.).

### Eugenia iantarensis

Taxon classificationPlantaeMyrtalesMyrtaceae

**Figure 13.**
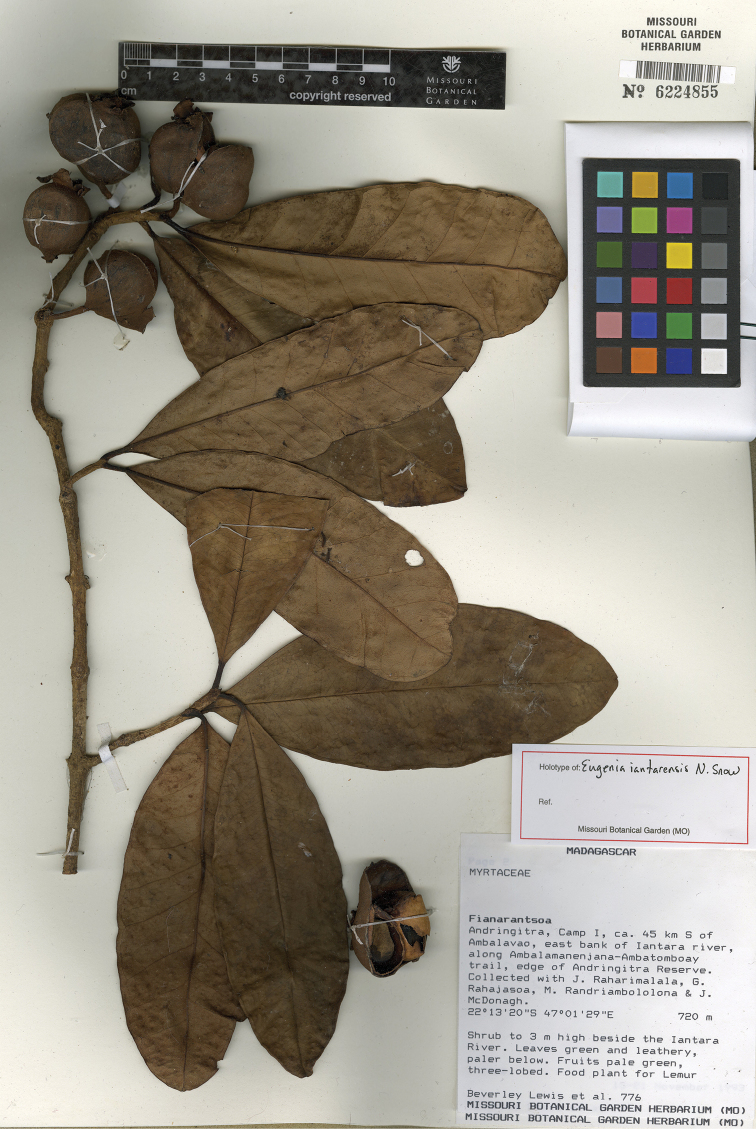
Holotype specimen of *Eugenia iantarensis* (MO).

N. Snow sp. nov.

urn:lsid:ipni.org:names:77146894-1

holotype (Figure 13): http://www.tropicos.org/Image/100314910; living material: http://www.tropicos.org/Image/100146017

Haec species a congeneris madagascariensibus foliis abaxialiter secus costae partem proximalem glandulis oleiferis manifestis ornatis, fructu permagno atque seminibus testa externa profunde sulcata convolutaque distinguitur.

#### Type.

MADAGASCAR. Prov. Fianarantsoa: Andringitra, Camp I, ca. 45 km S of Ambalavao, east bank of Iantara River, along Ambalamanenjana-Ambatomboay trail, edge of Andringitra Reserve, 22°13'20"S, 47°01'29"E, 20 m, 15–21 Nov. 1993, B. Lewis 776 + J. Raharimalala, G. Rahajasoa, M. Randriambololona & J. McDonagh (holotype: MO-6224855!]; isotypes: KSP [KSP000052]!, P, TAN).

#### Description.

Shrubs or trees, 3–9 m tall; dbh 6–10 cm; bark of main bole unknown. Vegetative and reproductive parts glabrous or (as noted) moderately to densely sericeous (trichomes dibrachiate and ferrugineous). Branchlets somewhat compressed laterally but becoming terete, becoming irregularly wrinkled in age, oil glands common, prominent and somewhat protruding initially but fading with age, grayish brown. Leaves concentrated mostly at branchlet tips, only 1–2 leaves produced during seasonal growth, the pairs ca. 1–1.5 cm distant; blades coriaceous, venation brochidodromous, surface and margin flat or slightly sinuous, somewhat discolorous, matte above and below. Axillary colleters absent. Petioles 6.5–10.5 mm long, rounded above, eglandular, shedding an outer (grayish or light brownish) epidermal layer with age. Leaf blades (9)11.5–22 × (3.4)5–8.7 cm, elliptic (or infrequently narrowly elliptic, narrowly obovate or obovate), base cuneate, apex obtuse; adaxial surface oil gland dense, easily seen with magnification, midvein sulcate but becoming flush apically, secondary veins protruding slightly; abaxial surface sparsely to moderately sericeous (hairs dibrachiate, ferrugineous) but becoming glabrous, oil glands common (notably less dense than adaxial), midvein protruding (and with punctate glands, especially proximally), punctate, striate, secondary veins somewhat protruding (moreso than on adaxial surface), intramarginal vein connecting tips of secondaries arching moderately, 1.5–3.3 mm from margin at laminar midpoint. Inflorescence of terminal, axillary or ramiflorous monads, solitary to paired or occasionally in few-flowered fascicles. Pedicels 8–21 mm long (in fruit), 1.8–3.5 mm wide, round in transverse section, rigidly stiff, ascending, sparsely hairy (use high magnification) or evidently glabrous, eglandular, somewhat striate, the epidermis reddish and eventually cracking in small irregular flakes, anthopodia and metaxyphylls lacking. Bracteoles (only one seen) evidently broadly triangular, ca. 2 mm long. Calyx lobes 4, 5.5–8.8 mm long, broadly rounded, apex obtuse, hairy towards based but otherwise more or less glabrous adaxially, moderately to densely hairy abaxially, bifacially glandular, persisting in fruit and tearing somewhat beneath the based as fruit matures, green to cream-colored or magenta above. Petals violet (Ratolojanahary 88). Staminal disk (in mature fruit) 7–16 mm wide, glabrous. Stamens (estimated from basal scars) 150+. Style (material scant) ca. 9 mm long, glabrous; stigma narrow (scarcely if at all capitate). Ovary 2-locular; placentation axile; ovules up to 5 per locule and radiating from central position. Berries 3.2–4.1 × (1.6)2.4–5.0 cm, irregularly globose and often with 3-numerous irregular lobes (from maturing seeds), densely shortly sericeous, pale green immature becoming grayish-brown when fresh but drying light rusty-brown by virtue of indumentum, outer wall embedded with numerous straight, more or less evenly spaced vertical veins extending between base and apex. Seeds 3–15 per fruit (up to 8 per locule), 17–25 mm long, up to ca. 15 mm thick (shrinking considerably away from seed coat during drying), radiating out from placenta (narrowed towards attachment point); seed coat thickly membranous to somewhat leathery, eglandular, separating easily from embryo, surface drying irregularly bullate, light reddish brown; cotyledons evidently completely fused.

#### Etymology.

The specific epithet is a Latinization of the Iantara River, location of the type gathering.

#### Vernacular names.

Rotramena vaventiravina (Kotozafy 922); rotra (Antilahimena 6914).

#### Phenology.

Flowering March through November; fruiting November and December.

#### Distribution.

Known from the eastern escarpment of Madagascar in Finarantsoa and Toamasina provinces around and in the Andringitra, Ranomafana and Analamazaotra Special Reserve (Fig. 11).

#### Habitat and ecology.

Evidently along or near rivers in humid forest, sometimes in disturbed areas; from 770–1210 m. The type collection indicates the plant, presumably the large fruits, is used by the Common brown lemur, *Eulemur fulvus*.

#### Conservation status.

With an EOO of 4,892 km^2^, an AOO of 81 km^2^ and three subpopulations, all of which are situated within the protected area network (Andringitra, Ranomafana and Analamazaotra), *Eugenia iantarensis* is assigned a preliminary status of “Least Concern” [LC] following the IUCN Red List Categories and Criteria (IUCN 2012).

#### Comments.

The flowering and early fruiting material (Toamasina Province) and mature fruiting material (Finarantsoa) are disjunct and the flowering material is somewhat incomplete (no petals), but apart from slightly shorter leaves in Toamasina the specimens match well.

Mature fruits are among the largest yet known among Malagasy *Eugenia*, and the large number of seeds per fruit (up to 13) is considerably greater than that commonly seen for *Eugenia* worldwide or in Madagascar. The testa in dried material becomes highly furrowed-convoluted to resemble the surface of a human brain, likely due only in part to shrinkage from dessication. A similar testal morphology was recently described in *Eugenia alletiana* Baider & Florens from Mauritius (Baider and Florens 2013), although the testa of that species at maturity was said to be as firm as the texture of wood. The selective pressures underlying the evolution of such a thick and tough testa are worthy of consideration in light of the observation on the holotype label that the trees are used by brown lemurs. The walls of the fruit and testa frequently have bore holes of an unknown insect of ca. 2 mm diameter.

#### Specimens examined.

MADAGASCAR. Prov. Fianarantsoa: Parc National de Ranomafana, 21°15'S, 47°25'E, 1045 m, 5 Dec. 2007, J. Renoult Ma07-225 (P [P05208539]); Parc National de Ranomafana, Parcelle II d’Ambodiamontana, Parcelle II de Talatakely, 21°16'S, 47°26'E, 950 m, 14 Mar. 1995, A. Kotozafy 922 (KSP [KSP000050, KSP000051], MO-04940615, P [P05208460]); Andringitra RN. Ivohibe, à l’W d’Ambarongy, au NE du campement no. 3, sur la piste à l’E du campement no. 3 vers le campement no. 4, 22°13'22"S, 46°58'18"E, 1210–1625 m, 8–11 Dec. 1994, C. Rakotovao 287 (MO-6224856, MO-5474705 [carpo.], P [P04884880]). Prov. Toamasina: Ampitambe, Ambatovy, Sahaviara forest, 18°52'19"S, 48°16'43"E, 1035 m, 9 Nov. 2005, P. Antilahimena 4143 & F. Edmond (MO, P [P05094984]); Ampitambe, Ambatovy, 18°51'39"S, 48°16'27"E, 943 m, 15 Dec. 2005, P. Antilahimena 4384 (MO, P [P00730616]); Ambatovy, Antaralava Besalampy forest, 18°53'15"S, 48°16'09"E, 950 m, 15 Nov. 2008, Antilahimena 6905 (MO-6447003, P [P00730615]); Ambatovy, Sahaevo, 18°50'26"S, 48°16'3"E, 982 m, 23 Nov. 2008, P. Antilahimena 6914 + B.A. Ratodimanana, D. Ravelonarivo, E. Félix & M. Ratovomanana (MO-6447031); Fkt: Ambohibolakely, forêt d’Amparihy, corridor Forestier Analamay Mantadia, 18°47'28"S, 48°22'46"E, 1001 m, 23 Apr. 2012, C. Rakotovao 5783 (P [P00730613]); Ampitambe, Ambatovy, forêt d’Ampadidifanantsy, 18°51'46"S, 48°16'32"E, 975 m, 27 Oct. 2008, M. Ratolojanahary 88 (MO-6453743, P [P04680790]); Forêt d’Analamazaotra, [18.56'S, 48.26'E], 3 Dec. 1934, Ursch 20 (P [P05208538]).

### Eugenia malcomberi

Taxon classificationPlantaeMyrtalesMyrtaceae

**Figure 14.**
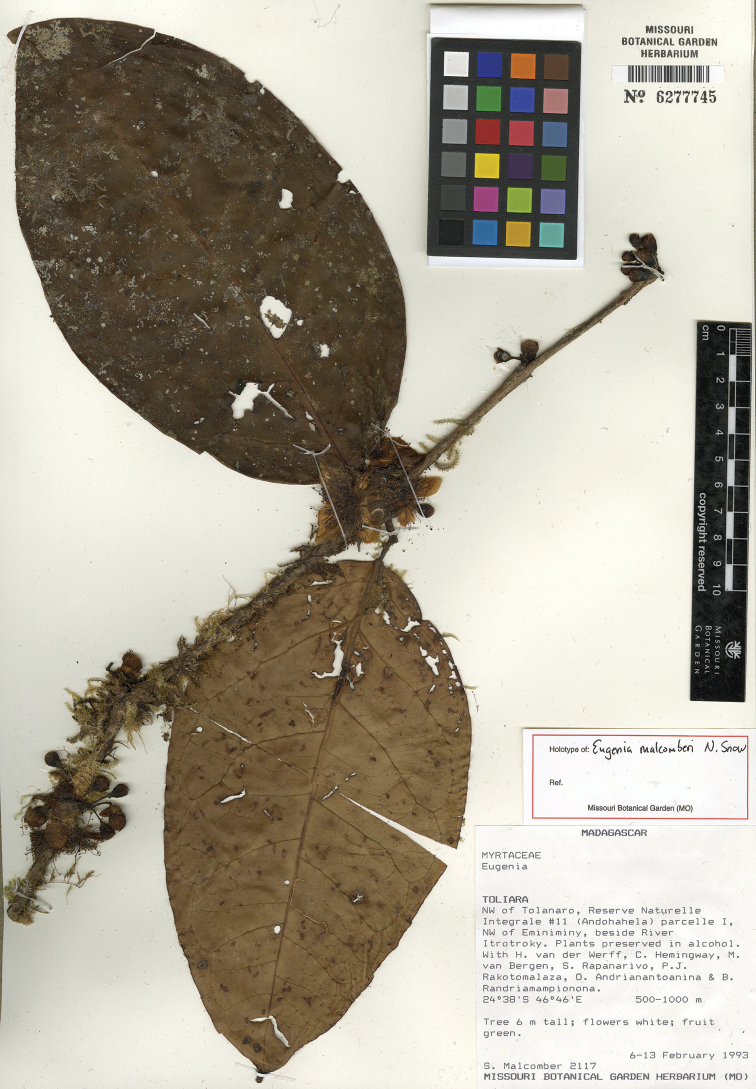
Holotype specimen of *Eugenia malcomberi* (MO).

N. Snow sp. nov.

urn:lsid:ipni.org:names:77146895-1

holotype (Figure 14): http://www.tropicos.org/Image/100314912

Haec species a congeneris madagascariensibus folia grandia habentibus foliis late ellipticis ad basem rotundatis plerumque plus quam 15 cm longis distinguitur.

#### Type.

MADAGASCAR. Prov. Toliara: NW of Tolagnaro, Reserve Naturelle Integrale #11 (Andohahela) parcelle I, NW of Eminiminy, beside River Itrotroky, 24°38'S, 46°46'E, 500–1000 m, 6–13 Feb. 1993, S. Malcomber 2117 + H. van der Werff, C. Hemingway, M. van Bergen, S. Rapanarivo, P.J. Rakotomalaza, O. Andrianantoanina & B. Randriamampionona (holotype: MO-6277745!; isotypes: KSP [KSP000046]!, P [P05131986]!, TAN).

#### Description.

Trees to 6 meters tall; bark of main bole unknown. Indumentum (where noted) of vegetative and reproductive material sericeous (hairs dibrachiate and typically reddish or brownish). Emerging (youngest) branchlets laterally compressed, sparsely hairy, glandular, color uncertain; older branchlets becoming terete, soon glabrous, mostly eglandular, becoming brownish-gray, smooth. Leaves evenly distributed along branchlets (nodes greater than 9 cm apart), coriaceous, venation brochidodromous, strongly discolorous, matte above and below. Axillary colleters present (type A; Snow et al. 2003) on youngest emerging leaves, otherwise absent. Petioles 8–11 mm long, flattened above, sparsely hairy upon emergence but becoming glabrous, glandular (particularly above adjacent to blade). Leaf blades 14.7–22.6 × (9.3–)10.0–)12.5 cm, broadly elliptic (mostly) or ovate, base rounded, margin and blade surface flat, apex and tip acute; abaxial surface sparsely hairy at emergence becoming glabrous, oil glands prominent (becoming less so), midvein broad (1.5–2.0 mm wide) and flush at emergence but becoming slightly sulcate in older leaves at least proximally; abaxial surface glabrous, oil glands sparse to moderate on laminar surface but common on midvein, midvein protruding but becoming flush or nearly so towards apex, secondary and intramarginal veins prominent, the veins connecting secondaries also prominent and broadly arching; intramarginal vein relatively faint, irregularly sinuous and 2–3 mm from midpoint at laminar margin. Inflorescence mostly ramiflorous or axillary, fasciculate clusters and comprised monads and triads to 5–7-flowered cymes. Bracteoles 1.1–1.8 mm × ca. 0.5 mm, ovate, sparsely hairy, glandular, mostly persisting during anthesis. Hypanthium 4.3–4.5 × 4.0–5.3 mm wide below base of calyx lobes, cupulate, sparsely to moderately hairy especially towards base, oil glands common to dense. Calyx lobes 4, 3.5–6.5 mm long, rounded to broadly obovate, apex obtuse, sparsely ciliate, oil glands moderately common and projecting (especially lower surface), consisting of two longer (inner) lobes and two shorter outer lobes (the bases of the outer lobes covering the bases of the inner lobes in bud), evidently whitish in flower. Petals 4 (5 on one flower), 11–17.5 × 6–12 mm, obovate to widely obovate, glabrous, white, oil glands sparse to common and pronounced. Staminal ring ca. 3.5–4.5 mm in diameter, glabrous. Stamens (estimated) 100–150; filaments 5–13 mm long; anther sacs 0.7–0.9 mm long, globose to subcylindrical; connective bearing one faint apical gland or eglandular. Ovary apex glabrous. Style 10–13 mm long, glabrous, eglandular; stigma narrow (barely if at all swollen). Berries not seen, indicated as being green on specimen label.

#### Epynomy.

The new species honors Dr. Simon Malcomber (b. 1967), who collected the type material and nearly two thousand specimens from Madagascar in the early 1990s.

#### Phenology.

Flowering in early February; fruiting probably late February through at least mid-March.

#### Distribution.

Known only from the type gathering in Toliara in parcelle I of the Reserve Naturelle Integrale #11 northwest of Taolognaro, in south-western Madagascar. This region is near the southern terminus of the humid forests that occur east of the main escarpment that run latitudinally along much of the length of Madagascar (Fig. 4).

#### Habitat and ecology.

The species was collected in a riverine habitat in a rainforest. Some of the (undistributed) type material is heavily clothed in epiphytic mosses, Hymenophyllaceae, and lichens.

#### Conservation status.

*Eugenia malcomberi* occurs in the Andohahela protected area (parcel I). No indication was provided on the label about its relative abundance at the time it was collected. Google™ Earth imagery (April 2013) shows a more or less continuous band of primary forest extending ca. 42 km south and ca. 135 km northeast, and ca. 10 km wide (east to west) at comparable elevations from the type locality. Considering its occurence in a nature reserve in fairly rugged terrain with considerable topographic relief, but also because it is known only from a single subpopulation with an AOO of 9 km^2^, *Eugenia malcomberi* is assigned a preliminary risk of extinction of “Vulnerable” [VU D2] following the IUCN Red List Categories and Criteria (IUCN 2012).

#### Comments.

The petals during anthesis are quite large relative to the size of the flower buds, suggesting that they enlarge rapidly during early anthesis.

### Eugenia manomboensis

Taxon classificationPlantaeMyrtalesMyrtaceae

**Figure 15.**
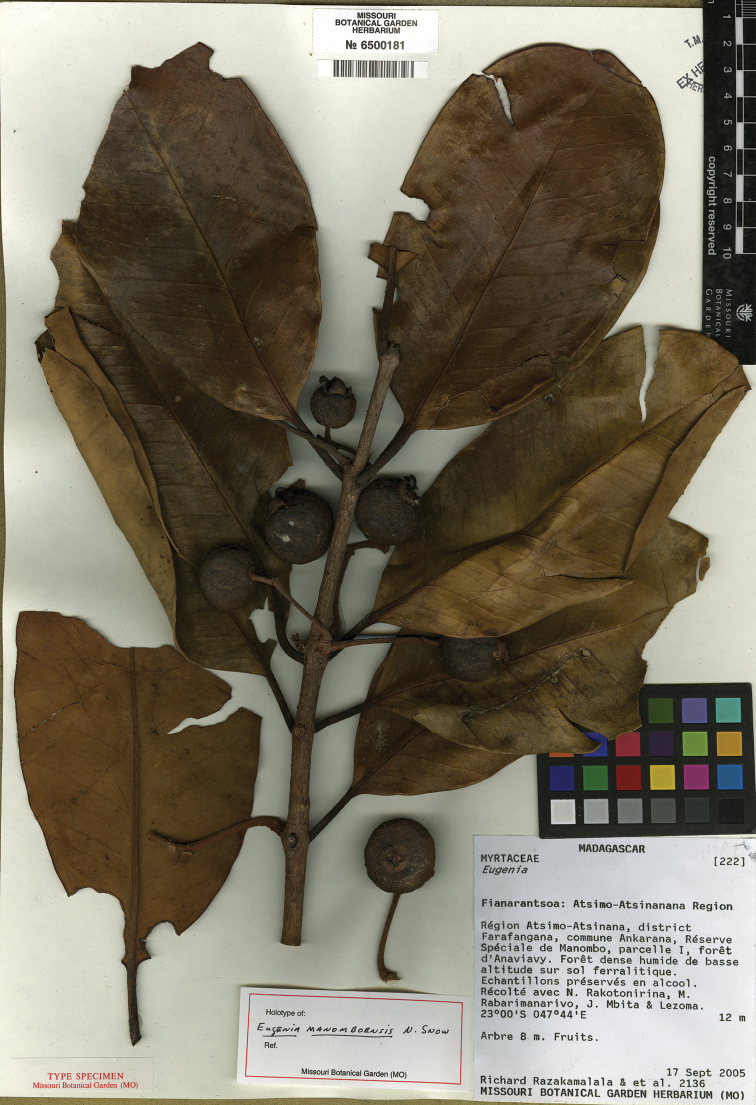
Holotype specimen of *Eugenia manomboensis* (MO).

N. Snow sp. nov.

urn:lsid:ipni.org:names:77146896-1

holotype (Figure 15): http://www.tropicos.org/Image/100317960

Haec species a congeneris madagascariensibus petiolo longo, lamina foliari grandi elliptica, pedicello crasso atque fructu grandi globulari laevi distinguitur.

#### Type.

MADAGASCAR. Prov. Fianarantsoa: Région Atsimo-Atsinanana, Dist. Farafangana, comm. Ankarana, Réserve Spéciale de Manombo, parcelle I, forêt d’Anaviavy, 23°00'S, 47°44'E, 12 m, 17 Sep. 2005, R. Razakamala 2136 (holotype: MO-2590168! isotypes: KSP [KSP000043]!, P [P04776395]!, TAN).

#### Description.

Trees to 8 m tall. Foliage glabrous and without evident oil glands except as noted. Branchlets round to somewhat laterally compressed, drying brown to light brown; bark smooth but cracking slightly longitudinally. Leaves stiffly coriaceous, opposite or sometimes three per node, evenly distributed along branchlets, discolorous, somewhat glossy adaxially, matte abaxially. Axillary colleters absent. Petioles 25–35 mm, terete or slightly sulcate. Leaves 16–18 × 6.5–10.5 cm, elliptic, base slightly cuneate to mostly rounded, apex obtuse, surface and margin more or less flat; adaxial surface with impressed midvein in lower ½ to 2/3, becoming flush distally; abaxial surface with dense but small oil glands (faint, use high magnification); secondary veins indistinct, arising at 40–50° angle but mostly too faint to count with confidence; intramarginal vein indistinct to barely visible, 1.5–2.5 mm from margin at midpoint of blade. Inflorescence up to 7 cm, structure uncertain but evidently of triads or botryoids, these solitary in leaf axils. Pedicels 4–14 mm, stiff and thick. Flowers mostly unknown. Calyx lobes (from dried fruit) 4, 3–5 mm, broadly rounded (much broader than long in fruit), persistent in fruit. Fruit 25–30 × 25–30 mm, globose.

#### Etymology.

The specific epithet is derived from the Réserve Spéciale de Manombo, the only known occurrence of this prominent species.

#### Phenology.

Fruiting mid September; likely flowering by August (unconfirmed).

#### Distribution.

Known only from the type gathering in south-eastern Madagascar in Fianarantsoa from Réserve Spéciale de Manombo, near the coast (Fig. 7).

#### Habitat and ecology.

Dense, humid, low-altitude forest over lateritic soils.

#### Conservation status.

*Eugenia manomboensis* occurs in the Réserve Spéciale of Manombo. Considering its occurrence in a natural reserve, but also because it is known only from single subpopulation (AOO of 9 km2), *Eugenia malcomberi* is assigned a preliminary risk of extinction of “Vulnerable” [VU D2] following the IUCN Red List Categories and Criteria (IUCN 2012).

#### Comments.

Large-leaved but indetermined speicmens of Malagasy *Eugenia* with large fruits have not been matched closely with flowering material in some cases. However, given that none of the larger-leaved species have petioles as thick or as long as *Eugenia manomboensis*, no others are known from near the type locality, and no large-leaved species present globular fruits of this size, it appears that this species has not been named previously.

It seems likely that the large fruits of this tree are consumed by local widelife.

### Eugenia obovatifolia

Taxon classificationPlantaeMyrtalesMyrtaceae

**Figure 16.**
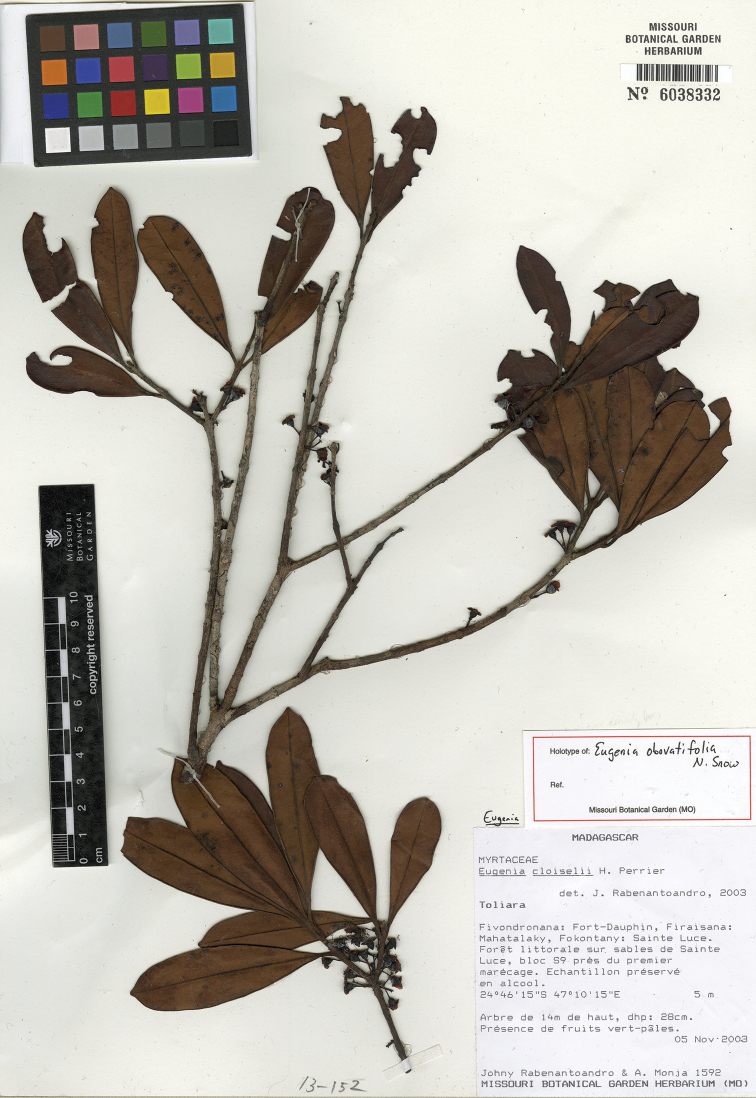
Holotype specimen of *Eugenia obovatifolia* (MO).

N. Snow sp. nov.

urn:lsid:ipni.org:names:77146897-1

holotype (Figure 16): http://www.tropicos.org/Image/100314904

Haec species a congeneris madagascariensibus foliis anguste obovatis atque inflorescentiis glabris saepe ramifloris distinguitur.

#### Type.

MADAGASCAR. Prov. Toliara: Fokontany: Sainte Luce, 24°46'15"S, 47°10'15"E, 5 m, 5 Nov. 2003, J. Rabenantoandro 1592 + A. Monja (holotype: MO-6038332!; isotypes: P [P04885376]!, TAN).

#### Description.

Trees 5–14 m; dbh 14–28 cm; bark of main bole unknown. Indumentum (where indicated) of short, ferrugineous, dibrachiate hairs that become increasingly white or grayish. Branchlets rounded laterally compressed, densely short-hairy becoming glabrescent; light brown to light gray (dried). Leaves concentrated near branch tips, coriaceous, surfaces matte. Axillary colleters absent. Petioles 4–8 mm, terete, pubescent when emerging. Leaf blades (3.0–)4.5–6.5 × 1.5–2.5 cm, narrowly obovate (or infrequently ovate); base narrowly cunate; apex obtuse or somewhat acute; margins flat but drying somewhat revolute; adaxial surface glabrous or moderately hairy (especially proximally), oil glands dense (but small; use magnification) on emergence but becoming less visible, midvein slightly sulcate to flush; abaxial surface moderately hairy, oil glands dense but faint; secondary veins 8–15, alternating along midvein, faint; intramarginal vein not visibile. Inflorescences ramiflorous, less than 1.5 cm long, solitary or more commonly fasciculate, of monads, triads, or short botryoids. Pedicels 1–5 mm, stiff, ascending, sparsely hairy to glabrous. Bracteoles 2, ovate, 0.5-1.0 × < 0.5 mm, rigid, sparsely hairy on margins. Hypanthium 2–3 mm, cuplate to obconic, glabrous to densely ferrugineous-hairy, densely glandular (glands relatively large). Calyx lobes 4, 1.5–2 mm, two each relatively large and small, broadly rounded (much broader than long), apex obtuse, sparsely minutely hairy marginally near apex, otherwise glabrous, greenish to cream colored. Petals 5–8 × 5–6 mm, elliptic to ovate, glabrous, whitish to pink or violet, oil glands sparse but easily visible. Stamens ca. 30–50, multiseriate; staminal disk short-hairy, ca. 1.5 mm diameter; filaments 3–5 mm, white; anthers ca. 0.5 mm, globular, basifixed, eglandular, light yellow. Styles 5–7 mm, glabrous; stigma narrow. Berries not seen, but labels indicate pale green (probably immature).

#### Etymology.

In reference to the shape of the leaf blades.

#### Vernacular name.

Ropsay (Rabenantoandro 366).

#### Phenology.

Flowering September, November, and January; fruiting August through December.

#### Distribution.

East-central Madagascar in Toamasina Province and south-eastern in Toliara Province from Mandena to Sainte Luce (Fig. 11).

#### Habitat and ecology.

Littoral forest over sand; elevation ca. 5–53 m.

#### Conservation status.

With an EOO of 8,333 km^2^, an AOO of 63 km^2^ and four subpopulations, none of which are situated within the protected network, *Eugenia obovatifolia* is assigned a preliminary risk of extinction of “Endangered” [EN 2ab(i, iii, iv)] following the IUCN Red List Categories and Criteria (IUCN 2012). The new species seems to be restricted to the highly threaten littoral forests and none of the known populations are currently protected.

#### Comments.

*Eugenia obovatifolia* has a general resemblance to *Eugenia arenicola* H. Perrier, *Eugenia hazompasika* H. Perrier, and *Eugenia cloiselii* H. Perrier given its ramiflorous, more or less fasciculate inflorescences. The four species also grow in relative proximity in southeastern and eastern Madagascar, often in sandy littoral forests, and may form a species complex that ranges approximately 1300 km between Taolognaro and Sainte Luce along the coast and somewhat inland to Vatomandry.

*Eugenia obovatifolia* differs from *Eugenia arenicola* by its more or less glabrous leaves, whereas those of the latter are densely ferrugineous-hairy upon emergence and retain much of the indumentum on the abaxial surface. *Eugenia obovatifolia* differs each from *Eugenia cloiselii* and *Eugenia hazompasika* by virtue of the glabrous (or nearly so) inflorescences, which contrast with the hairy inflorescences of the latter two.

Several indetermined specimens cannot be confidently placed yet given variation in leaf morphology and indumentum (e.g., Razanatsima 31 + Ranaivojaona; Ranaivojaona 1177 + Razanatsima; Razanatsima 711 + Céléstin; Rakotovao 3755 et al. [all at MO]). However, including all of these taxa and specimens into one highly heterogenous species likely would equate to the inappropriate lumping of several independently evolving lineages.

#### Specimens examined.

MADAGASCAR. Prov. Toamasina: Fokontany: Andravokoditra, 18°34'53"–18°34'20"S, 49°14'42"–49°14'54"E, 7 Jan. 2003, R. Ludovic 228 & F. Rakotoarivony (MO-6038324); Hotel Pangalan on Lake Ampitabe, west of Andranokoditra, 18°35'S, 49°14'E, S.K. Pell 607 + J.D. Mitchell & A. Randrianasolo (MO-5887243); Fokontany: Andranonkoditra Vohibola du côté de l’hôtel Pangalane, 18°35'32"S, 49°14'02"E, 11 Feb. 2003, J. Rabenantoandro 1248 + R. Rabevohitra, P. Lowry, R.Razakamalala & S. Lowry (MO-6038325); Ambinaninony, Andranokoditra, Akinin’ny nofy, 18°34'12"S, 49°14'18"E, 3 Aug. 2003, R. Razakamalala 664 & D. Rabehevitra (MO-6038323); Ambila-Lemaitso, 18°49'S, 49°08'E, 14 Dec. 1967, Service Forestier 28034 (B, BR, G, K, MO-6321087, NY, WAG). Prov. Toliara: Mandena, Jardin Botanique, M16-QMM, Ampasy, Taolagnaro, 24°57'05"S, 47°00'11"E, 26 Sep. 2000, J. Rabenantoandro 241 + P. Lowry, R. Rabevohitra, L. Randrihasipara, & E. Ramisy (MO-5598572); Saint-Luce (Manafiafy), S9, Mahatalaky, Taolagnaro, 24°46'30"S, 47°10'20"E, 16 Dec. 2000, J. Rabenantoandro 366 + F. Lucien & E. Ramisy (MO-5728973); Manambaro, Petriky, 25°03'43"S, 46°52'06"E, 15 Nov. 2006, Ramison 33 (MO-6427490).

### Eugenia ranomafana

Taxon classificationPlantaeMyrtalesMyrtaceae

**Figure 17.**
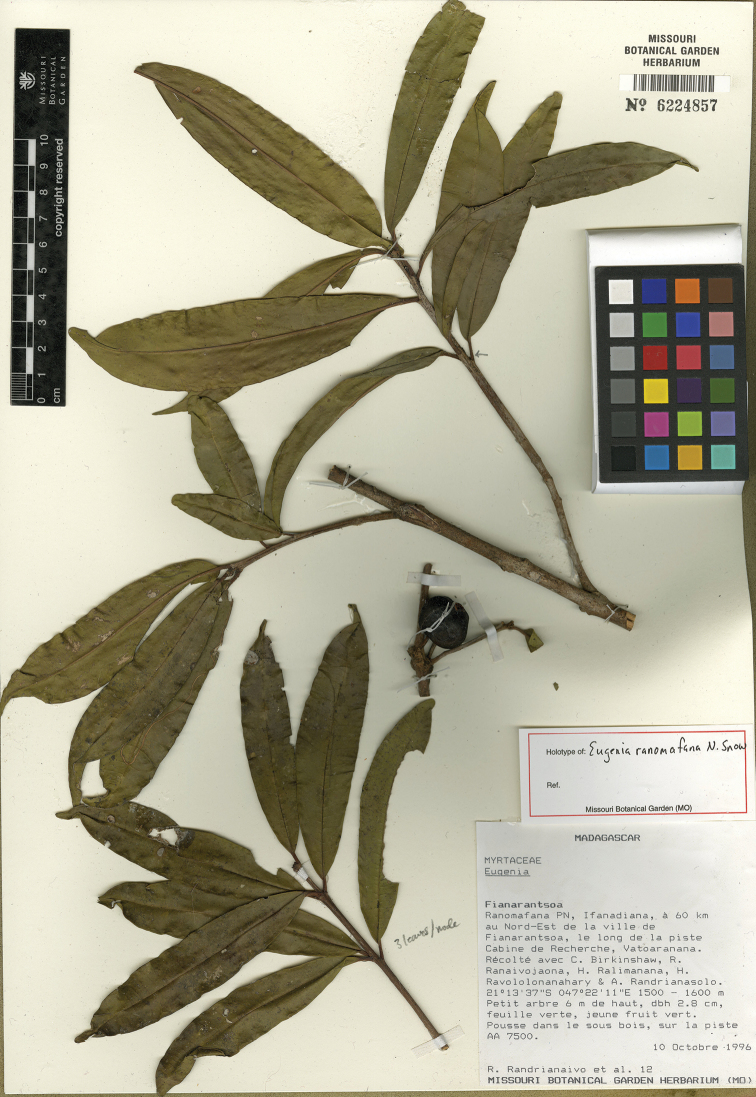
Holotype specimen of *Eugenia ranomafana* (MO).

N. Snow & D. Turk sp. nov.

urn:lsid:ipni.org:names:77146898-1

holotype (Figure 17): http://www.tropicos.org/Image/100314914

Haec species a congeneris madagascariensibus ramulis in sectione transversali non profunde sed late sulcatis, foliis saepe 3- vel 4-verticillatis atque hypanthio sub anthesi discoideo distinguitur.

#### Type.

MADAGASCAR. Prov. Fianarantsoa: Ranomafana PN, Ifanadiana, à 60 km au Nort-Est de la ville de Fianarantsoa, le long de la piste Cabine de Recherche, Vatoaranana, 10 Oct. 1996, 21°13'37"S, 47°22'11"E, 1500–1600 m, R. Randrianaivo 12 et al. (holotype: MO-6224857!; isotypes: KSP [KSP000044, KSP000045]!; P [P04885335]!, TAN).

#### Description.

Shrubs or trees, 3–8 m tall; dbh 2.8 cm; bark of main bole unknown. Foliage and flowers glabrous except as noted below. Branchlets laterally compressed to terete but sometimes trigonal in cross section, and irregularly and broadly and prominently sulcate on one or more sides, flaring distally below nodes but becoming rounded; smooth but sometimes striate, sparsely serciceous (trichomes reddish and dibrachiate) but soon glabrous, epidermis reddish or maroon on emergence but becoming irregularly and finely brown-gray mottled (dried material). Leaves opposite to disjunct opposite or whorled in 3s or 4s, concentrated near branch tips; coriaceous; venation brochidodromous; surfaces concolorous, matte; lateral veins 20–32 on each side, arising at an angle of 65–75˚ from the midrib. Axillary colleters lacking. Petioles 3–7(–12) mm, deeply sulcate adaxially, prominently striate-rugose (especially abaxially), eglandular. Leaf blades (4.4– )6.0–16.0 × (1.9)2.8–3.3 cm, narrowly elliptic, often conduplicate (dried material) in lower ca. 20%, base mostly rounded (sometimes cuneate), apex obtuse but sometimes slightly falcate, margin and surface somewhat sinuous; adaxial midvein deeply sulcate throughout, eglandular; abaxial surface eglandular except on midvein (glands moderately dense and somewhat indistinct); secondary veins consisting of (16–)25–32 pairs, indistinct but slightly raised, diverging at wide angle from midvein; intramarginal vein indistinct, 1.5–2.5 mm from margin of leaf at midpoint of blade. Inflorescence of solitary, paired, or fasciculate and ramiflorous monads. Pedicels (4.5–)8–17 mm, stiff but thin, terete to slightly compressed. Bracteoles 2, 0.5–0.7 × ca. 0.2–0.5 mm, broadly ovate, apex rounded, horizontal to reflexed in fruit, occasionally a few short trichomes. Hypanthium 0.5–1.5 mm long; initially cupuliform but becoming discoid (prominently flattened from above) during and after anthesis, glabrous, oil glands common, cream-colored (fresh). Calyx lobes 4 (but of unequal lengths), the two shorter lobes broader than long, ca. 1.5 × 2.2 mm broad at base, the two longer lobes hemispherical, ca. 2 × 2 mm, glandular, pale green (fresh), persisting or deciduous in fruit, ascending or crowing mature fruit. Petals 4, ca. 4 mm × 3.2 mm, broadly obovate to broadly elliptical, pink, with 9 veins arising from the base. Staminal disk diameter (in fruit) 3.5–5.0 mm; glabrous. Stamens ca. 60–80; filaments (1.5–)4–6 mm, cream-colored; anther sacs ca. 0.5 mm, subglobose to globose, connective apex eglandular; staminal disk glabrous. Style ca. 4–6 mm; stigma narrow. Fruit 11–18 × 8–23 mm, subglobose with irregular lobes (but not costate), base rounded, green (young fresh) but drying dark bluish-black, locules 2, placenta capitate, placentation axile, ovules radiating from center of placenta. Seeds 3–4 per fruit; outer coat leathery, highly contorted, drying to a light yellowish-brown (“fawn”) or dull brownish-yellow (“tawny”, Beentje 2010).

#### Etymology.

From Ranomafana National Park.

#### Phenology.

Fruiting October and November; flowering May.

#### Distribution.

Known from three collections in Ranomafana National Park in Fianarantsoa Province, southeast-central Madagascar (Fig. 4).

#### Habitat and ecology.

Growing in the undergrowth or subcanopy in humid forests, ca. 950–1600 m. The collection by Turk notes its occurrence with trees of the genera *Weinmannia* L., *Tambourissa* Sonn., *Decarydendron* Danguy, *Ephippiandra* Decne., *Ocotea* Aubl. and *Cryptocarya* R. Br., and understory with *Psychotria* L. and *Oncostemum* A. Juss.

#### Conservation status.

With only three collections known, an AOO of 9 km^2^ and one subpopulation, which is situated within the protected area network (Ranomafana), *Eugenia ranomafana* is assigned a preliminary risk of extinction of “Vulnerable” [VU D2] following the IUCN Red List Categories and Criteria (IUCN 2012).

#### Comments.

This species was first recognized as undescribed by Turk (1997) as part of a dissertation studying plants in Ranomafana National Park, who approved of and collaborated with its publication here.

With its narrowly elliptic leaf blades with mostly rounded leaf bases, *Eugenia ranomafana* resembles mostly closely *Eugenia wilsoniana*, the nearest occurrence of which is ca. 280 km northeast. *Eugenia wilsoniana* differs by its shorter stature, leaf margins being flat to only slightly sinuous, longer and thinner (on average) pedicels, presence of axillary colleters, and a hairy staminal disk. In addition, *Eugenia ranomafana* differs by its often trigonous branchlets and the frequent occurrence of 3–4 leaves per node.

#### Specimens examined.

MADAGASCAR. Prov. Fianarantsoa: Ranomafana National Park, parcelle 3, S of National Road 25 at 7 km W of Ranomafana; Talatakely trail system, 21°15'30"S, 47°25'00"E, 950–1150 m, 4 May 1993, D. Turk 436 + J. Randrianasolo, J. Solo, & D. Randriamanatenta (MO, KSP); Ranomafana National Park, Talatakely parcel, Trail System F-TBT-B, 21°15'S, 47°25'E, P. Fritsch 1643 et al. (CAS, MO-6287616).

### Eugenia ravelonarivoi

Taxon classificationPlantaeMyrtalesMyrtaceae

**Figure 18.**
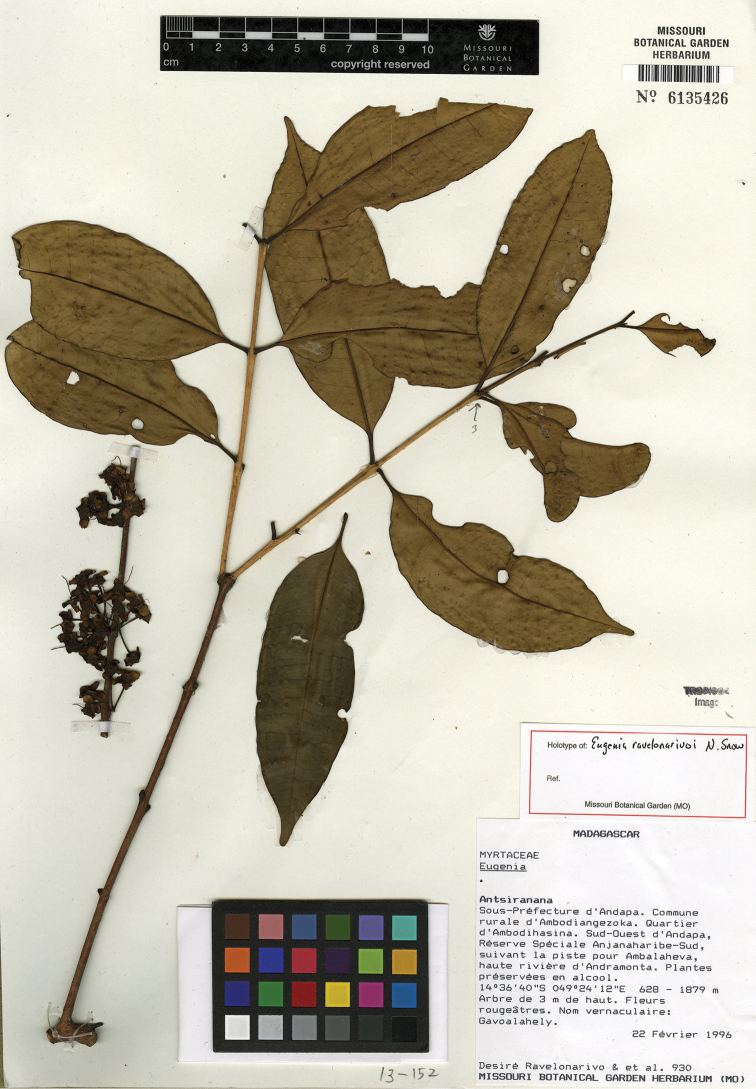
Holotype specimen of *Eugenia ravelonarivoi* (MO).

N. Snow & Callm. sp. nov.

urn:lsid:ipni.org:names:77146899-1

holotype (Figure 18): http://www.tropicos.org/Image/100314919

Haec species a congeneris madagascariensibus foliis apice abrupte cupidatis caudatisve atque inflorescentiis ramifloris dense fasciculatis distinguitur.

#### Type.

MADAGASCAR. Prov. Antsiranana: SW d’Andapa, Réserve Spéciale Anjanaharibe-Sud, suivant la piste pour Ambalaheva, haute rivière d’Andramonta, 14°36'40"S, 49°24'12"E, 628–1879 m, 22 Feb. 1996, D. Ravelonarivo et al. 930 (holotype: MO-6135426!; isotypes: KSP [KSP000007, KSP000008]!, P [P04885351]!, TAN).

#### Description.

Shrubs or trees, 3–12 meters; bark of main bole unknown. Herbage glabrous except as noted. Branchlets laterally comprssed and shallowly 2-grooved on each side when young below petioles, oil glands common but indistinct, epidermis smooth, green drying light brown. Leaves evenly distributed along branchlets, opposite to disjunct opposite, thinly coriaceous to thickly membranous, discolorous, surfaces matte. Axillary colleters absent. Petioles 7–10 mm, narrowly and deeply sulcate (especially distally). Leaf blades (5.5–)7.5–14 × 3.3–4.8 cm, mostly elliptic but some broadly elliptic or narrowly oblong, base cuneate, apex obtuse, or acuminate and often falcate, margins flat but slightly undulate (dried); adaxial surface midvein deeply but narrowly sulcate to nearly the apex, secondary veins numerous but faint, oil glands absent; abaxial surface lakcking oil glands, secondary veins faint, slightly raised, diverging at ca. 80°; intramarginal vein 0.5–1.5 mm from margin at midpoint of blade. Inflorescences of moderately dense fascicles of monads arising from short brachyblasts, these evidently ramiflorous or cauliflorous (uncertain given that all are detached on herbarium sheets); pedicels 9–15 mm, rigid, irregularly glandular. Bracteoles 2, ca. 0.5–0.8 mm, triangular to ovate, often obscure. Hypanthium ca. 2–2.5 mm, cupuliform to discoid (somewhat flattened) in anthesis, prominently glandular. Calyx lobes 4, 2.0–5.0 mm, broadly oblong to oblate or rounded, often dimorphic (2 shorter, 2 longer), apex broadly rounded, petaloid, oil glands sparse (and mostly abaxial). Petals 4 (material scant), up to 5 mm; indicated as pinkish on a paratype. Staminal ring more or less square, ca. 4 mm per side, sparsely short hairy (use magnification) and glandular. Stamens (material scant) numerous; anthers ca. 0.5 mm, ellipsoid. Styles 6–11 mm, thin, sparsely glandular (especially proximally); stigma narrow. Berries 24–35 × 30–50 mm (dried), subglobular, greenish (mature or nearly so); seeds 1–3, 16–22 × 25–30 mm, subglobular; embryo globular, hypocotyl and cotyledons not differentiated; testa thinnish, drying light brown.

#### Phenology.

Flowering January through April; fruiting March through November.

#### Distribution.

In the northern mountains of Madagascar in Antsiranana and Mahajanga provinces (Fig. 4).

#### Habitat and ecology.

In mid-elevation, wet eastern forests often over granite or gneiss; elevation approximately 630–1880 m but needing confirmation.

#### Etymology.

It is a pleasure to name this species in honor of Désiré Ravelonarivo (b. 1966), a prolific collector of specimens in Madagascar. Désiré is native to the Andapa basin, where he collected the type specimen of *Eugenia andapae* and the only known fruiting specimen of this new species (D. Ravelonarivo 489 & R. Babesonina).

#### Vernacular name.

Gavoalehely (Ravelonarivo 930); gavoala (Ravelonarivo 103).

#### Conservation status.

With an EOO of 436 km^2^, an AOO of 36 km^2^ and three subpopulations, two of which are situated within the protected area network (Anjanaharibe-Sud), *Eugenia ravelonarivoi* is assigned a preliminary risk of extinction of “Vulnerable” [VU D2] following the IUCN Red List Categories and Criteria (IUCN 2012).

#### Comments.

The label of the type gathering indicates “rougeâtres” (reddish) for petals, but this is doubtful and probably was meant for pinkish, which accords with information on the label of a paratype.

Among existing species, the leaf morphology of *Eugenia ravelonarivoi* resembles that of *Eugenia alatroensis* H. Perrier and *Eugenia vatomandrensis* H. Perrier, but the bases of their leaves are subsessile to sessile, which contrasts with the distinctly petiolate leaves of *Eugenia ravelonarivoi*. In addition, the often cuspidate-caudate aspect of the leaf apex of new species is much more pronounced than of those two species, whose tips are barely (if at all) cuspidate.

*Eugenia antongilensis* H. Perrier has cuspidate apices on the blade, but it is a much longer leaf with shorter petioles. *Eugenia musicola* H. Perrier also resembles *Eugenia ravelonarivoi*, especially given the membranous to weakly coriaceous texture of the leaf blades; however, the pedicels of the former are much thinner and more lax, and its petioles are shorter and much less (if at all) sulcate adaxially. *Eugenia diospyroides* differs with its generally larger flowers and leaves, and leaves having a rounded apex.

The new species also closely resembles *Eugenia radiciflora* in leaf morphology, but that species is said to have solitary flowers with pedicels only 2–3 mm long, which does not match the fasciculate brachyblasts of *Eugenia ravelonarivoi*. The leaves of *Eugenia ravelonarivoi* also superficially resemble those of *Eugenia gandhii*, but the latter has densely punctate leaf blades and solitary flowers. Finally, *Eugenia ravelonarivoi* resembles closely some evidently undescribed taxa

Dr. David Gordon, an entomologist at Pittsburg State University, indicates that the visible damage on the leaves of some specimens may be from leaf cutter bees (Family Megachilidae), which often damage leaves in this manner to obtain material for the construction of their nests.

#### Additional specimens.

MADAGASCAR. Prov. Antsiranana: SW d’Andapa, Réserve Spéciale d’Anjanaharibe-Sud, aux enrivons de sommet, 14°46'15"S, 49°28'00"E, 1112–1424 m, 21 Mar.–7 Apr. 1994, Ravelonarivo 103 + F. Rasoavimbahoaka, B.T. Rafaliarimanana, H. Rasitefarinina & Motera (P [P04884879]); Massif de l’Anjanaharibe (pentes et sommet N) a l’W d’Andapa (Haut Andramonta, Bassin de Lokoho), [14°37'S, 49°25'E], 900 m, 10 Dec. 1950–9 Jan. 1951, H. Humbert 24576 + R. Capuron & G. Cours (P [P05208600]); Sud-Ouest d’Andapa, Réserve Spéciale d’Anjanaharibe-Sud, village d’Andranotsarabe, suivant la route Nationale d’Andapa-Bealanana de lat piste vers à l’oueste, Ambatoomainty, Camp No. 2, 14°44'22”S, 49°27'42”E, 3 Nov. 1994, 1185–1335 m, D. Ravelonarivo 489 & R. Babesonina (KSP [KSP003115, KSP003116], P [P05208456]). Prov. Mahajanga: Ankaizinana, [14°30'S, 48°55'E], 1400 m, 20 Apr. 1923, R. Decary 2009 (P [P00118106]).

### Eugenia razakamalalae

Taxon classificationPlantaeMyrtalesMyrtaceae

**Figure 19.**
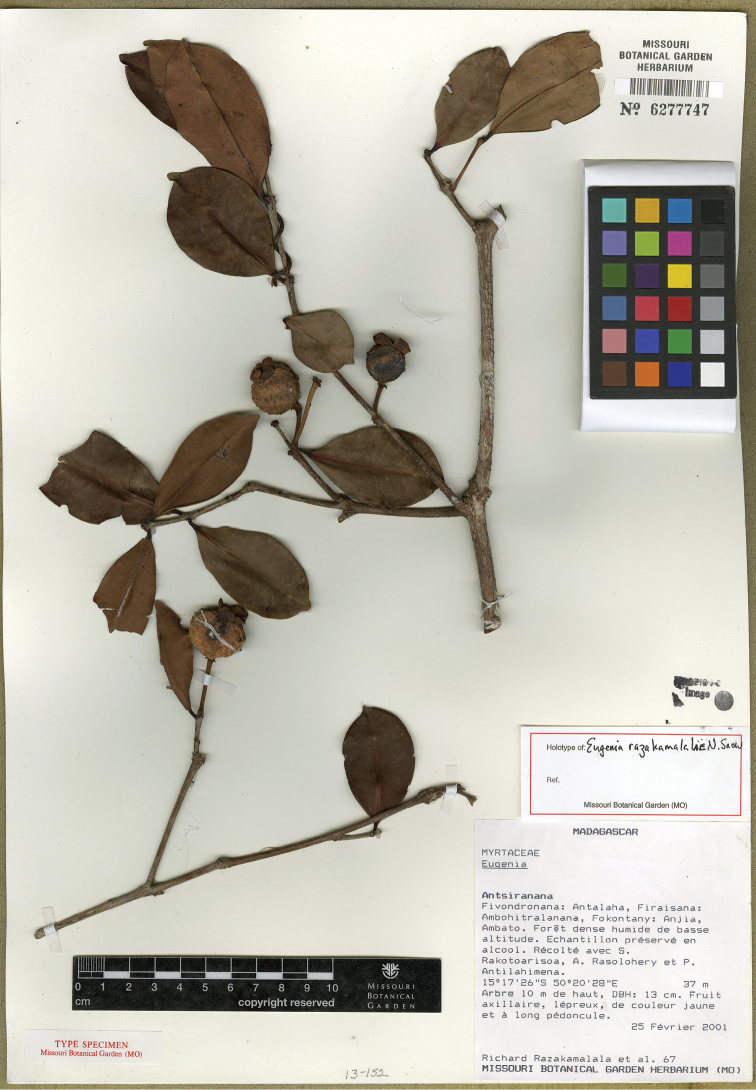
Holotype specimen of *Eugenia razakamalalae* (MO).

N. Snow & Callm. sp. nov.

urn:lsid:ipni.org:names:77146900-1

holotype (Figure 19): http://www.tropicos.org/Image/100314920

Haec species a congeneris madagascariensibus lamina foliari rigide coriacea late elliptica usque obovata utrinque ad paginas (nec ad margines tantum) nonnihil sinuosa atque glandulis densis folii paginam abaxialem pedunculos hypanthium lobos calycinos petala necnon fructum ornantibus distinguitur.

#### Type.

Madagascar. Prov. Antsiranana: Fokontany: Anjia, Ambato, 15°17'26"S, 50°20'28"E, 37 m, 25 Feb. 2001, R. Razakamalala 67 + S. Rakotoarisoa, A. Rasolohery & P. Antilahimena (holotype: MO-6277747!; isotypes: KSP [KSP000003]!; P, TAN).

#### Description.

Trees to 10 m; dbh up to 13 cm; bark of main bole unknown. Plants glabrous throughout. Branchlets laterally compressed; youngest epidermis drying light brown to light gray but soon peeling and flaking to reveal reddish-brown color (dried); oil glands of epidermis prominent and common. Leaves stiffly coriaceous, mostly concentrated near branch tips, medium green and slightly discolorous, irregularly sinuous, surfaces matte. Axillary colleters absent. Petioles 3–5 mm, becoming slightly sulcate distally and broadening towards apex. Leaf blades 3.5–9.0 × 2.5–5.0 cm, broadly elliptic to obovate or broadly obovate, base cuneate, apex obtuse to somewhat acute; adaxial surface with midvein broadly but shallowly sulcate in the lower half, becoming flush distally; oil glands absent; abaxial surface oil glands common to dense but small and faint and fading with maturity (visible only with magnification), secondary veins somewhat indistinct, intramarginal vein indistinct and ca. 0.5–1.0 mm from margin at midpoint of blade. Inflorescence terminal or axillary, monads 1–3 in each axil. Pedicels 15–25 mm, relatively thick (1.2–2.0 mm at maturity), ascending to erect, rigid. Bracteoles 2, 1–2 mm, ovate, stiff, persistent and more or less divergent in fruit. Hypanthium 3.5–5 mm, cupulate, oil glands common to dense. Calyx lobes 4, 5–8 mm, broadly rounded, apex obtuse, densely glandular, persistent and crowning the fruit, stramineous or rufous-beige (fresh). Flowers unknown. Berries 1.5–1.8 × 2.0–2.3 cm, subglobular to globular, densely glandular, slightly verrucose (dried), yellowish (possibly not fully mature).

#### Etymology.

It is a pleasure to name this species in honor of Richard Razakamalala (b. 1962), a Malagasy botanist who has collected over seven thousand specimens of plants from Madagascar since 2001. His prolific collecting efforts have contributed significantly to our increased knowledge of the Malagasy flora. Four of his *Eugenia* collections are designated as type specimens in this publication.

#### Phenology.

Fruiting late February.

#### Distribution.

Known only from the type gathering in Antsiranana Province, north-eastern Madagascar, about 14 km west of the coast in the Masoala Peninsula (Fig. 2).

#### Habitat and ecology.

In dense humid lowland forests over quartzite from ca. 35 m elevation.

#### Conservation status.

With only one collection known from just outside the Masoala protected area, *Eugenia razakamalalae* is assigned a preliminary risk of extinction of “Endangered” [EN 2ab(i, iii, iv)] following the IUCN Red List Categories and Criteria (IUCN 2012). It is highly probable that the new species occurs within Masoala National Park, and is therefore protected.

#### Comments.

The leaf morphology *of Eugenia razakamalalae* resembles that of Eugenia arthroopoda H. Perrier var. ambalavensis H. Perrier, an invalid name given the lack of a Latin diagnosis in the protologue (Perrier de la Bâthie 1953a). However, the pedicels, hypanthium and calyx lobes of Humbert 22,804 (a syntype of var. ambalavensis) have a dense, uniform, appressed rusty indumenta that is also present on the outer surface of the petals, apart from an approximately 1 mm wide glabrous margin. In contrast, the pedicels in fruit of *Eugenia razakamalalae* are glabrous (flowering material is unknown).

Snow (2011) discussed some morphological characters of the type gathering of *Eugenia razakamalalae* relative to those of *Eugenia lacerosepala* N. Snow, the latter of which is known only from its type gathering ca. 300 km north-northwest. The two species are similar in having fruits that are borne atop stiff and relatively long pedicels that are prominently and densely glandular, in having a slightly verrucose texture, and in being crowned by prominent calyx lobes. However, the narrowly obovate and shorter (2.1–3.5 cm) leaves of *Eugenia lacerosepala* are quite unlike those of *Eugenia razakamalalae*.

### Eugenia richardii

Taxon classificationPlantaeMyrtalesMyrtaceae

(Blume) N. Snow, Callm. & Phillipson comb. nov.

urn:lsid:ipni.org:names:77146902-1

Isotype: http://sonneratphoto.mnhn.fr/2012/08/23/1/P00118150.jpg

#### Basionym.

*Jossinia richardii* Blume in Mus. Bot. 1: 123. 1851.

#### Type.

MADAGASCAR. Prov. Antsiranana: forêt à Vohémar, [13°21'30"S, 50°00'30"E], comm. 1837, J.M.C. Richard 119 (holotype: L [L0009467]!; isotype: P [P00118150]!).

#### Description.

Shrubs 2–4 m tall; ca. 4 cm dbh; bark of main bole rough, grayish-brown. Branchlets terete, lightish gray-brown (dried); internodes short, (0.5–)1.5–2.3 cm; epidermis smooth but becoming fissured and flaking on older internodes, indumentum moderately dense of minutely dibrachiate trichomes; oil glands common, and somewhat prominent, and protruding above surface. Leaves mostly evenly distributed along branchlets, coriaceous, slightly discolorous, surfaces matte. Axillary colleters absent. Petioles 1–2 mm, slightly sulcate but mostly flush adaxially, glabrous. Leaf blades 2.5–4.5 × 1.6–2.5 cm, elliptic; base rounded to somewhat cuneate; apex broadly obtuse to rounded; margins slightly revolute; adaxial surface glabrous, oil glands dense upon emergence but fading as blade thickens, midvein broadly but slightly sulcate proximally; abaxial surface resembling adaxial, secondary veins indistinct or not visible, intramarginal vein 1.0–1.5 mm from margin at midpoint of blade. Inflorescence a monad arising in terminal leaf axils. Anthopodia (short internode between base of hypanthium and subtending bracteoles) present, 1–2 mm. Pedicels 1.0–1.4 mm, firm but slightly bowed, glabrous. Bracteoles 2, 0.8–1.0 × ca. 1.0 mm, ovate, minutely hairy on margins near apex. Hypanthium 4–5 mm, cupuliform to obconic, glabrous but evidently exuding salt, oil glands common to dense; ovary apex densely short-hairy. Calyx lobes 4, ca. 4 × 4 mm (material limited), oblong, apex obtuse, glabrous, greenish. Petals 9–10 × 6–9 mm, widely elliptic to widely obovate, sparsely short-ciliate on margins, thin (nearly transluscent), pinkish, oil glands sparse to common but prominent. Stamens ca. 70–100, multiseriate; anthers ellipsoid, 0.6–0.8 mm, sub-basifixed, connective with a single apical gland. Filaments 3–9 mm, white; ovary apex densely short-hairy. Styles 9–10 mm, glabrous; stigma narrow. Berries unknown.

#### Phenology.

Flowering August; fruiting January and likely commencing in late August (unconfirmed).

#### Distribution.

Known from the north-eastern coastal region of Madagascar in Antsiranana Province, from Orangéa to about 140 km along the coast near Vohemar (Fig. 2).

#### Habitat and ecology.

Littoral forests over sand, inland from the ocean shore less than 1 km; elevation 10 m.

#### Conservation status.

With an EOO of 358 km^2^, an AOO of 36 km^2^ and three subpopulations, one of which is situated within a proposed protected area that currently holds a temporary protection status (Oronjia), *Eugenia richardii* is assigned a preliminary risk of extinction of “Endangered” [EN B1ab(iii)+ B2ab(iii)] following the IUCN Red List Categories and Criteria (IUCN 2012). The species seems to have a highly restricted distribution in dry deciduous forests over sand. The area around Oronjia is mostly degraded as a result of human activities and is under serious threat. A definitive protection of the dry deciduous forests over sand of Oronjia would likely enable the species to be downlisted from the current risk of extinction to “Vulnerable”.

#### Comments.

*Eugenia richardii* most closely resembles two species, including *Eugenia vanwykiana* N. Snow, which differs by its larger leaves with cuneate bases (Snow et al. 2012). The second fairly close match is *Eugenia calciscopulorum*, which occurs significantly farther inland (ca. 11 km) than *Eugenia richardii* and at a higher elevation (ca. 410 m) and over limestone, compared to the sandy substrates of *Eugenia richardii*. The leaves of *Eugenia calciscopulorum* have more pronounced secondary veins above and below, retain the pronounced glands into the mature leaf stage, and have a more pronounced petiole.

*Eugenia richardii* also resembles *Eugenia quadriflora* H. Perrier, which is known from a single collection in 1961 from east of Lake Alaotra. Both species have thickly coriaceous, densely glandular leaf blades with irregularly undulating surfaces and moderately to strongly recurved margins, and simple flowers arising terminally. However, the leaves of *Eugenia quadriflora* are sessile with strongly and irregularly recurved blades (although this may reflect to some degree its condition when pressed and dried); in contrast, the leaf blades of of *Eugenia richardii* are distinctly but shortly petiolate and slightly recurved.

Elsewhere, the leaf morphology of *Eugenia richardii* somewhat resembles that of *Eugenia neofasciculata* Bennet from Mauritius (formerly *Eugenia fasciculata* Guého & A.J. Scott). Based on comparison digitally with an isotype (Lorence DL 1214 (P [P00390098]!), the species are similar by virtue of stiffly coriaceous, densely punctate, obovate-elliptic leaf blades with slightly undulating surfaces and recurved margins that arise from relatively short petioles. However, whereas the adxial petiolar surface of *Eugenia richardii* is flat, that of *Eugenia neofasciculata* is somewhat sulcate. In addition, the inflorescence of *Eugenia richardii* is a terminal pair of flowers with one arising in each leaf axil, whereas that of *Eugenia neofasciculata* is conspicuously fasciculate (mostly) on naked branches or within leaf axils.

#### Specimens examined.

MADAGASCAR. Prov. Antsiranana: Vohémar, [12°16'24"S, 49°23'20"E], s.d., L.H. Boivin 2697 (=J.M.C. Richard 134) (P [P00118153]); Orangéa, [12°14'S, 49°22'E], 1–100 m, 22 Jan. 1960, G. G. Cours 5394 (P [P05097489]; Env. de Diégo-Suarez, Orangéa, [12°14'S, 49°22'E], 1–100 m, 22 Jan. 1960, H. Humbert 32245 + G. Cours (P [P05576991]); Ramena, Baie des Sakalava, 3.5 km à l’E du Fokontany Ankorikihely, 12°16'24"S, 49°23'20"E, 17 Aug. 2004, J.B. Leopold 27 + L.J. Razafitsalama, R. Guittou, J. Be, & V. Benjara (CNARP, KSP [KSP000005], MO-6245567, P [P05208542], TAN); Andrafiabe, presqu’île entre Ambolobozobe et Ambolobozokely, 12°29'39"S, 49°34'04"E, 9 Feb. 2005, F. Ratovoson 965 + R. Guittou & D. Elifara (G, MO-6028974, P [P05208572]); Vohémar, [13°21'30"S, 50°00'30"E], s.d., J.M.C. Richard 75 (P [P00118148, P00118149]).

### Eugenia tiampoka

Taxon classificationPlantaeMyrtalesMyrtaceae

**Figure 20.**
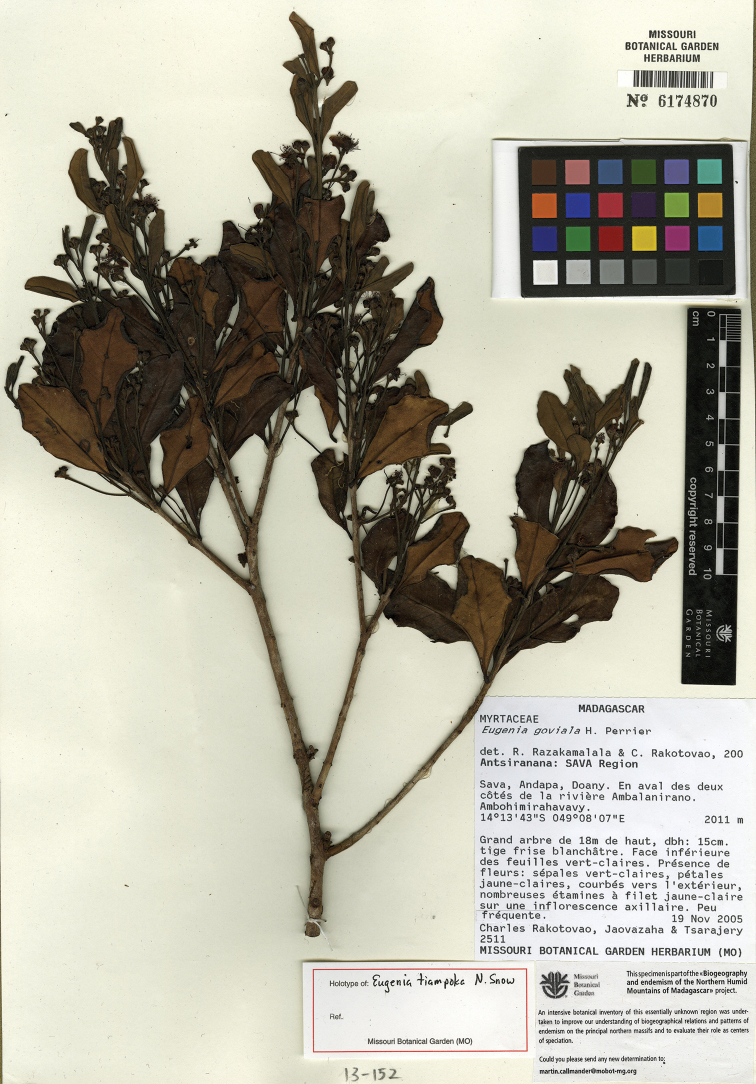
Holotype specimen of *Eugenia tiampoka* (MO).

N. Snow & Callm. sp. nov.

urn:lsid:ipni.org:names:77146901-1

holotype (Figure 20): http://www.tropicos.org/Image/100314918; Living material: http://www.tropicos.org/Image/76837

Haec species Eugeniae thouvenotianae H. Perrier simillima, sed ab ea lamina foliari glandulis oleiferis punctatis abaxialiter ornata atque pedicellis multo brevioribus distinguitur.

#### Type.

MADAGASCAR. Prov. Antsiranana: Doany, en aval des deux côtes de la rivière Ambalanirano. Ambohimirahavavy, 14°13'43"S, 49°08'07"E, 2011 m, 19 Nov. 1995, C. Rakotovao 2511 + Jaovazaha & Tsarajery (holotype: MO-6174870!; isotypes: G [G00341423]!, P [P05260190]!, TAN).

#### Description.

Trees 8–18 m; dbh 12–60 cm; bark of main bole unknown. Indumentum of foliage and floral parts dibrachiate, short and appressed, ferrugineous, denser upon emergence but becoming (often) nearly glabrous (or as indicated below). Branchlets rounded to laterally compressed, greenish fresh but drying light brown to light gray; epidermis thin and peeling away in thin strips or flakes, oil glands absent to sparse and indistinct. Leaves discolorous, margin sometimes strongly revolute and irregularly sinuous, venation reticulate, emerald and somewhat glossy adaxially, citrine and matte abaxially, concentrated near branch tips. Axillary colleters occasionally present. Petioles (2.5–)5–10, narrowly and deeply sulcate adaxially, elgandular. Leaf blades 4.0–6.8 × 2.3–.8 cm, elliptic to obovate, base cuneate, apex obtuse or occasionally retuse; adaxial surface remaining at least sparsely sericeous when fully emerged (trichomes closely appressed), oil glands common to dense but faintly visible, midvein deeply and narrowly suclate proximally becoming flush distally; abaxial surface indumentum as above, oil glands not as dense but much more prominent, secondary veins indistinct to somewhat prominent, intramarginal vein 1.0–1.5 mm from margin at midpoint of leaf blade (but often obscured by revolute margin). Inflorescence 2–5 cm long, mostly axillary or ramiflorous, sometimes terminal, consisting of triads, botryoids, or less commonly metabotryoids or pseudo-umbels of 4–5 flowers apically clustered on peduncle; peduncles mostly solitary but often opposite in leaf axils or on naked branches, mostly ascending and somewhat stiff but also sometimes flexuous. Pedicels 1–2 mm long. Bracteoles absent; bract-like structures surrounding triads of flowers when inflorescence is emerging but these soon deciduous. Hypanthium 1.5–2.5 mm, obconic, densely hairy and densely punctate. Calyx lobes 4, (1.2–)1.5–1.8 mm, broadly rounded, apex obtuse, mostly glabrous above to sparsely sericeous, moderately sericeous dorsally and on margins below, deciduous in fruit, light green when fresh. Petals 4, 4–7 mm, obovate, minutely and sparsely ciliate apically (use magnification) but otherwise glabrous, oil glands sparse to moderate. Stamens 20–45, exserted; staminal disk glabrescent; ovary apex glabrous; filaments 4–7 mm, yellowish-white; anthers 0.4–0.6 mm, globose to subelliptic, basifixed, eglandular; style 4–5 mm, stigma narrow and scarcely if at all capitate. Fruit (material limited), ca. 2.2 × 2.2 cm, globose, glabrate, green when young drying nearly black; outer layer leathery and prominently glandular. Seed 1 at maturity (material scant), round from above but laterally compressed (due to pressing?), ca. 16 × 16 × 9 mm; embryo not differentiated into distinct cotyledons or epicotyl.

#### Etymology.

The specific epithet is derived from elements of the native language of northern Madagascar, in which *ampoka* means mosses. *Eugenia tiampoka* thus means the “*Eugenia* that likes mosses”.

#### Phenology.

Flowering October through February; fruiting by early April, likely commencing by March and extending through May.

#### Distribution.

Northern Madagascar in Antsiranana Province in the northern mountains (Fig. 2).

#### Habitat and ecology.

Mid- to high-altitude humid forests laden with mosses, sometimes along river banks; 1990–2300 m in the northern highlands of Madagascar. A team of botanists lead by MC conducted a series of field expeditions between 2005 and 2008 to explore the flora and vegetation of this long-neglected region, during which more than 4,400 collections were made, yielding material of several plant species new to science (Callmander et al. 2008, 2009, 2012).

#### Conservation status.

With an EOO of 71 km^2^, an AOO of 45 km^2^ and two subpopulations, neither of which is within the protected area network, *Eugenia tiampoka* is assigned a preliminary risk of extinction of “Endangered” [EN B1ab(iii)+2ab(iii)] following the IUCN Red List Categories and Criteria (IUCN 2012).

#### Comments.

The leaves reportedly are aromatic (Birkinshaw 950, Rakotovao 2398). One specimen is said to have a nectariferous disk (Randriarivelo 350). A specimen of 30 m stature (Ravelonarivo 647) from the same general area and also said to be aromatic, but growing at 1700 m, has a slightly different aspect to the leaves, also may be this species.

The morphological gestalt of the leaves and inflorescences resemble most closely those of *Eugenia thouvetiana* H. Perrier, which however lacks the punctate oil glands of the abaxial leaf surface, and which has much longer pedicels subtending the individual flowers than those of *Eugenia tiampoka*. The type locality of *Eugenia thouvetiana* is located in Analamazaotra-Andasibe in Toamasina Province, some 525 km south of the southernmost known collection of *Eugenia tiampoka*. *Eugenia tiampoka* also somewhat resembles *Eugenia hazompasika* H. Perrier, the latter of which has much longer, broader and stiffer leaves with a less abruptly cuneate base.

#### Specimens examined.

MADAGASCAR. Prov. Antsiranana: 13 km N of Mangindrano, Tsaratanana Massif, SW ridge of Andomanisambiraro, 14°08'41”S, 48°57'53”E, 2150 m, 17 Oct. 2001, C. Birkinshaw 950 + R. Lala (KSP [KSP000048, KSP000049], P [P05156041]); Crête E d’Ambohimirahavavy, 14°12'15”S, 49°05'54”E, 2278 m, 3 Nov. 2005, C. Rakotovao 2398 + Jaovazaha & Torize (G, MO-6202095); Sommet de Beampoko, Ambohimirahavavy, 14°13'55”S, 49°08'23”E, 2137 m, 21 Nov. 2005, C. Rakotovao 2566 + Jaovazaha & Tsarajery (G, MO-6174671, P [P05260203]); Doany, forêt d’altitude d’Ambohimirahavavy, 14°13'41”S 49°08'14”E, 1991 m, 18 Nov. 2005, C. Randrianarivelo 350 + J. Randriantiavina & Torize (G, MO-6081166), KSP [KSP000047], P [P05260189]); Fokontany: Ampanompy, Ampanompy, 14°08'31”S, 48°58'04”E, 2050–2300 m, 5 Apr. 2001, R. Razakamalala 99 + R. Ranaivojaona, F. Ratovoson, A. Rasolohery, A. Andriamaniry & Mahavory (KSP [KSP000047]!).

### Eugenia wilsoniana

Taxon classificationPlantaeMyrtalesMyrtaceae

**Figure 21.**
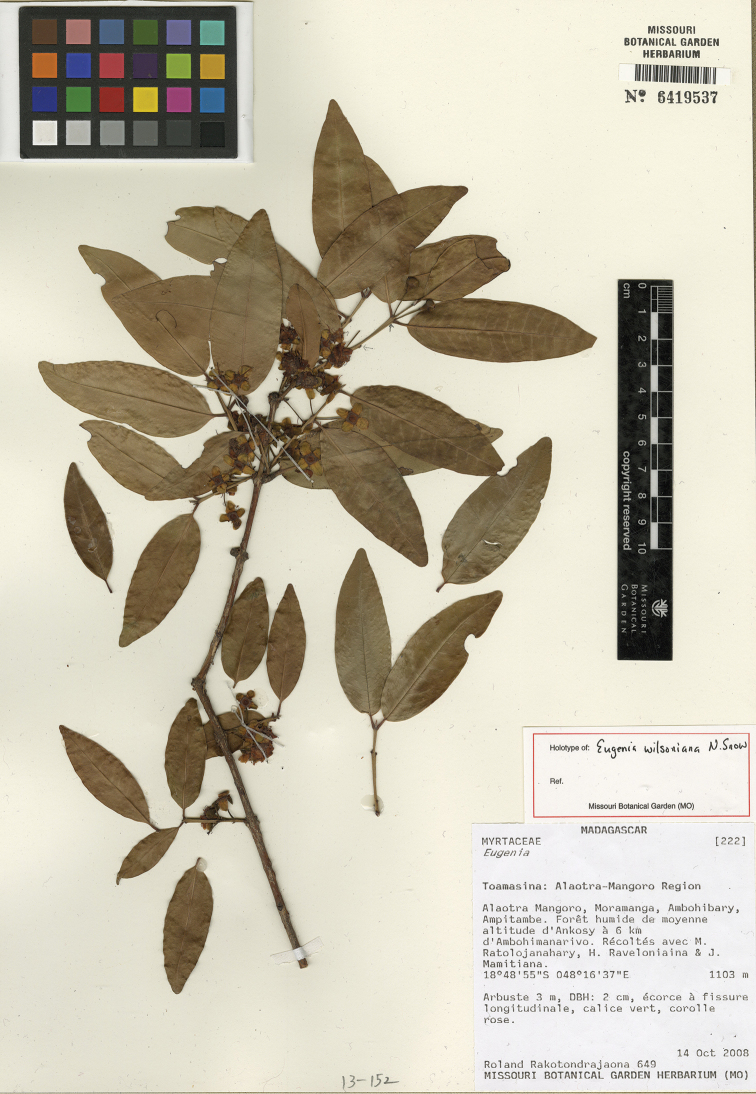
Holotype specimen of *Eugenia wilsoniana* (MO).

N. Snow sp. nov.

urn:lsid:ipni.org:names:77146903-1

holotype (Figure 21): http://www.tropicos.org/Image/100314905

*Haec species* Eugeniae bemangidiensi N. Snow *simillima, sed ab ea inflorescentia axillari longipedicellata distinguitur; etiam ad altitudines superiores crescit.*

#### Type.

MADAGASCAR. Prov. Toamasina: Alaotra Mangoro Reg., Moramanga, Ambohibary, Ampitambe, 18°48'55"S, 48°16'37"E, 1103 m, 14 Oct. 2008, R. Rakotondrajaona 649 (holotype: MO-6419537!); isotypes: P, TAN).

#### Description.

Shrubs or trees, (1–)2–6 m. Trunk dbh ca. 2 cm (measurements few); bark of main bole fissured, maroon. Indumentum where present of highly asymmetric and short, ferrugineous, dibrachate hairs. Branchlets laterally compressed, smooth, sparsely puberulous, drying light brown to greenish, punctate glands absent. Leaves opposite or disjunct opposite, mostly concentrated near tips of branches; venation reticulate; blades thinly coriaceous, dark green above and lighter green below. Axillary colleters obscure when present. Petioles 3–5 mm, deeply and narrowly sulcate adaxially, laterally compressed, elgandular, glabrescent (especially abaxially), longitudinally striate initially but thickening and becoming somewhat latitudinally striate with age. Leaf blades 4.0–12.5 × 1.4–3.0 cm, narrowly elliptic to narrowly ovate, base rounded and slightly constricted-conduplicate above petiole, apex acute, margins flat; adaxial surface glabrous, eglandular, midvein narrowly but deeply sulcate lower 2/3–4/5; abaxial surface like adaxial except: midvein protruding, sometimes prominently glandular and longitudinally striate, secondary veins faint to prominent, arising only 10–20° from midvein, tertiary veins faint to nearly as prominent as secondaries; intramarginal vein 1.0–1.5 mm from leaf edge at midpoint of blade. Inflorescence terminal, axillary, or arising on naked branch of current year’s growth. Flowers solitary, or occasionally as up to four arising from short brachyblasts; pedicels 6–12 × ca. 0.5 mm, glabrous, strongly laterally compressed (especially distally), longitudinally striate, sparsely to moderately glandular. Bracteoles 2, 0.6–1.1 mm, narrowly ovate to ovate, sparsely hairy abaxially and apically, rigid, somewhat ascending to appressed against base of hypanthium. Hypanthium 2.5–4.0 mm, cupuliform, sparsely hairy becoming glabrous, sparsely to moderately (but only faintly) glandular. Calyx lobes 4, 4–5 × 3–5 mm, oblate to broadly elliptic, apex broadly obtuse to rounded, minutely and sparsely ciliate in upper half, prominently glandular, greenish when fresh. Petals 4, 4–5 × 2–3 mm, widely elliptic to ovate, sparsely short-ciliate upper 1/2–1/3, faintly and somewhat sparsely glandular, whitish or pinkish. Stamens exserted, staminal disk ca. 4 mm diameter, more or less square from above, short-hairy, adjacent ovary apex glabrous; filaments 2–3 mm; anthers 0.8–1.0 mm, elliptic, yellow, sub-basifixed, apical gland between connectives lacking. Styles 7–8 mm, glabrous; stigma narrow and scarcely if at all capitate. Berries (immature?) 12–15 × 12–15 mm, globular, greenish.

#### Etymology.

In honor of Dr. Peter G. Wilson (b. 1950) of the Royal Botanic Gardens in Sydney, Australia; colleague, collaborator, and long-time student of Myrtaceae (e.g., Wilson and Waterhouse 1982; Wilson et al. 2005; Snow and Wilson 2010; Wilson 2011).

#### Vernacular name.

Hazompasina (Antilahimena 4935); Rotra (Ravelonarivo 3148).

#### Phenology.

Flowering mid-October through mid-November; fruiting October and November.

#### Distribution.

East-central Madagascar in Toamasina Province, occurring near Analamazaotra National Park (Fig. 2).

#### Habitat and ecology.

In humid, sometimes disturbed forests at middle altitudes from ca. 980–1103 m.

#### Conservation status.

With five collections known, an AOO of 9 km^2^ and one subpopulation that is situated outside the protected area, *Eugenia wilsoniana* is assigned a preliminary risk of extinction of “Critically Endangered” [CR A3c+B1ab(iii)] following the IUCN Red List Categories and Criteria (IUCN 2012). Current data suggest the species has a narrow distribution, and the montane evergreen tropical forests where the species grows are threatened by mining activities. The discovery of this species in one of the nearby protected areas probably would allow downlisting to “Endangered”.

#### Comments.

The leaf and floral morphologies of *Eugenia wilsoniana* are suggestive of *Eugenia diospyroides* H. Perrier. However, *Eugenia wilsoniana* has much shorter leaves and a hairy staminal ring, in contrast with the much larger leaves and glabrous staminal ring of *Eugenia diospyroides* (e.g., Randriatifika 118 et al. [KSP]). This new species resembles even more closely another newly described species herein, *Eugenia bemangidiensis*, which differs by its cauliforous inflorences occurring in tight, relatively short fascicles, secondary veins on the leaf blades arising at steeper angles, and occurring in a different habitat at much lower elevations some 750 km southeast from the presently known occurrences of *Eugenia wilsoniana*. Moreover, the foliage and floral parts of *Eugenia bemangidiensis* are entirely glabrous, the abaxial laminar midvein protrudes less prominently, the adaxial laminar sulcus is not as deep or narrow, and its adaxial petiolar sulcus is broader and shallower than those of *Eugenia wilsoniana*.

#### Specimens examined.

MADAGASCAR. Prov. Toamasina: Ambatovy, Sahaevo forest, 18°50'26"S, 48°16'33"E, 11 Nov. 2006, P. Antilahimena 4935 & F. Edmond (MO-6338238); ibid. loc., 18°50'26"S, 48°16'34"E, 23 Nov. 2008, P. Antilahimena 6912 + B.A. Ratodimanana, D. Ravelonarivo, E. Félix & M. Ratvomanana (MO-6447030); Ambatovy, Antaniditra, 18°49'11"S, 48°16'53"E, 13 Oct. 2008, R. Bernard 1154 (MO); Ampitambe, Ambatovy, 18°49'06"S, 48°17'06"E, 1041 m, 13 Oct. 2008, R. Rakotondrajaona 642 + M. Ratolojanahary (MO-6419531); Mararano, Marovoay, 18°48'10"S, 48°17'59"E, 14 Nov. 2008, D. Ravelonarivo 3148 (MO-6447573).

### Lectotypifications

Many species of *Eugenia* in Madagascar described by Perrier de la Bâthie (1953a) eventually will require lectotypification to establish coherent species concepts and avoid nomenclatural confusion. We have reviewed all of the existing published small-leaved *Eugenia* species in Madagascar and the currently available specimens, and have seen some additional material that has helped to clarify species delimitations. Here we lectotypify three names to help clarify species’ boundaries and nomenclature.

Lectotypification is particularly important for *Eugenia scottii*, since the protologue included twelve syntype collections that clearly included more than one taxon. *Eugenia hovarum* H. Perrier and *Eugenia scottii* H. Perrier were described for relatively small-leaved species occuring mostly in the mountainous parts of Madagascar, and both were based on multiple syntypes (Perrier de la Bâthie 1953a).

***Eugenia scottii*** H. Perrier in Mém. Inst. Sci. Mad., Sér. B, Biol. Vég. 4(2): 168-169. 1953. **Lectotype** (designated here): MADAGASCAR. Prov. Antsiranana: Massif du Tsaratanana et haute vallée du Sambirano, Mt. Andilabe, 2300 m, Nov.-Dec. 1937, H. Humbert 18501 (lectotype: P [P00118145]!; isolectotype: P [P00118146]!).

Perrier de la Bâthie (1953a: 169) referred to *Eugenia scottii* as having a large number of forms that he could not distinguish taxonomically (“…*un très grand nombre de formes que nous n’avons pu distinguer*”). In light of the material now available, we believe that the specimens can be assigned comfortably to five different species, four of which already have valid names. Among the syntypes, we have chosen Humbert 18501 at 2300 m elevation from the Tsaratanana Massif in Antsiranana Province as the lectotype because it is among the better flowering syntype collections, and represents a species that cannot be assigned to any other published taxon. None of the syntypes at P were annotated specifically by Perrier de la Bâthie as the type, or designated as such in his original description or subsequently in the Flora treatment (Perrier de la Bâthie 1953b). Humbert 18501 comprises two sheets at P, so we designate the slightly more copious of the two (P [P00118145]), which bears an original hand-written label. as the lectotype. The other specimen with a printed label (P [P00118146]), as an isolectotype. As we now circumscribe *Eugenia scottii*, it is restricted to mountain summits and ridges in ericoid shrubland and rocky areas, mostly over 2000 m a.s.l. on Tsaratanana and Marojejy. It includes two other original syntypes of *Eugenia scottii*: Humbert 18389 and Humbert + Cours 23709.

Among the specimens that we exclude from *Eugenia scottii* is material that can be accommodated in *Eugenia cassinoides* Lam., a species from the littoral forest in the south-east (Perrier de la Bâthie 6486); *Eugenia richardii* (e.g., Boivin 2697, Richard 75, Richard 119), a species from low elevation dry forest in the north-east (the needed new combination of which is provided in this article); *Eugenia petrikiensis* N. Snow & Randriat. (Scott Eliott 2847) from Fort Dauphin; and a new, as yet undescribed species from the mountains of the south-east at mid elevation in the Mandrare basin (Humbert 6503, 6685, 6835), and probably also includes the last syntype, Humbert 11965, from Kalambatritra.

**Additional specimens examined:** MADAGASCAR. Prov. Antsiranana: Réserve Naturelle Intégrale du Marojejy, versant W et SW du sommet de Marojejy, 14°27'S, 49°44'E, 2040 m, 15 Nov. 1996, Rakotomalaza 918 et al. (G, MO, P [P05208453]); Massif du Tsaratanana, hauts sommets de l’Amboabory à l’Antsianongatalata, 2300-2609 m, Nov.-Dec. 1937, H. Humbert 18389 (P [P00118147]); Sommet oriental du Massif du Marojejy, à l’W de la Haute Manantenina, affluent de la Lohoho, 1850-2137 m, 26 March–2 Avril. 1949, H. Humbert 23709 + G. Cours (P [P00118154]).

**Excluded syntypes:** MADAGASCAR. Prov. Antsiranana: Vohemar, L.H. Boivin 2697 (= J.M.C. Richard 134) (syntype: P [00118153]!); Vohémar, [12°16'24"S, 49°23'20"E], s.d., J.M.C. Richard 75 (syntypes: P [P00118148, P00118149]!); Vohémar, [12°16'24"S, 49°23'20"E], comm. 1837, J.M.C. Richard 119 (syntypes: L [L0009467]!, P [P00118150]!). Prov. Fianarantsoa: massif de Kalambatitra (centre S), Mt. Analatsitendrika, Nov. 1933, H. Humbert 11965 (syntypes: P [P00118151, P00118152]!); près de l’embouchure du Matitana (SE), Oct. 1911, H. Perrier de la Bâthie 6486 (syntype: P [P00118144]!). Prov. Toliara: Haute vallée du Mandrare (SE), 600-900 m, 8–15 Nov. 1928, H. Humbert 6503 (syntypes: P [P00118141, P00118142]!); Bassin sup. du Mandrare (SE), du col de Vavara à la vallée du Manambolo, 700–1200 m, 20–22 Nov. 1928, H. Humbert 6685 (syntype: P [P00118140]!); Bassin sup. du Mandrare (SE), Mt. d’Amboahangy près d’Esira, 1000–1150 m, 25 Nov. 1928, H. Humbert 6835 (syntypes: P [P00118139, P00118138]!); Fort Dauphin, s.d., Scott Eliott 2847 (syntype: P [P00118137]!).

***Eugenia hovarum*** H. Perrier in Mém. Inst. Sci. Mad., Sér. B, Biol. Vég. 4(2): 170. 1953. **Lectotype** (designated here): MADAGASCAR. Prov. Antananarivo: env. de Manjakandriana, comm. 1932, Perrier de la Bâthie 14007 (lectotype: P [P00118032]!; isolectotype: P [P00118031]!). **Syntype:** MADAGASCAR. Prov. Antananarivo: Imerina, comm. 20 Nov. 1889, Campenon s.n. (P [P00118033]!).

Perrier de la Bâthie (1953a: 170) only cited two syntypes that clearly belong to species that we consider to be *Eugenia hovarum*. Not atypically, Perrier de la Bâthie made no indication of which should be regarded as the holotype, but his own collection (Perrier de la Bâthie 14007) is the more complete of the two, comprises two separate sheets at P, and bears a detailed collection locality information, unlike the other syntype, and is here designated as the lectotype.

**Additional specimen examined:** MADAGASCAR. Prov. Antsiranana: Parc National de Ranomafana, ca. 4 km E of Vohiparara, 21°13'32”S 47°26'43”E 1170 m, 5 Nov. 1993, Schatz 3531 (MO, P [P05208543]).

***Eugenia nompa*** H. Perrier in Mém. Inst. Sci. Mad., Sér. B, Biol. Vég. 4(2): 181. 1953. **Lectotype** (designated here): MADAGASCAR. Prov. Antsiranana: Dist. Ambanja, Beangona (haut Sambirano), [14°03'00"S 48°42'15"E], 24 Oct. 1947, Réserves Naturelles 1275 (lectotype: P [P00118008]!; isolectotype: P [P00118007]!). **Syntypes:** MADAGASCAR. Prov. Antsiranana: env. du Mt. Tsaratanana, Nov. 1912, Perrier de la Bâthie 5481 (P [P00118004, P00118005, P00118006]!).

The species concept for *Eugenia nompa* H. Perrier has been uncertain. Because the syntypes were clearly designated by Perrier de la Bâthie (1953a), we lectotypify one of these to clarify its diagnostic characters, which include: leaf blades narrowly elliptic, apex acute; midvein deeply and narrowly sulcate lower 75%+; margins somewhat sinuous; inflorescence of somewhat elongate cymes or sparsely-flowered botryoids, terminal or axillary in clusters, the whole (at least when young) covered with a ferrugineous indumentum.

Perrier de la Bâthie (1953a) also described Eugenia nompa var. arborea H. Perrier, an invalid name based on Perrier de la Bâthie 16261. The inflorescence structure of those specimens differ considerably from the lectotype recognized above, and it is still unclear whether specimens designated as this variety can be confidently placed in extant taxa.

**Additional specimen examined:** MADAGASCAR. Prov. Antsiranana: District Ambanja, Canton: Marovato. Réserve Naturelle 4 (Tsaratanana), 21 Sept. 1953, Réserve Naturelle 5750 (P [P05097466]).

## Discussion

The eighteen species of *Eugenia* newly proposed here increases the known number of native species in Madagascar from approximately 46 to 68, all of which are endemic. Of these, 32 (approximately half) have been described in the last fifteen years (Miller 2000; Snow 2008, 2011; Snow et al. 2012 and this paper). Specimens of the introduced and possibly naturalized Neotropical species *Eugenia uniflora* L. and *Eugenia brasiliensis* Lam. have been confirmed by the first author (housed at MO). Both species produce edible fruits, and the former also is used as a hedgerow. The paucity of collections suggests that these species are not widely planted.

Many of the original descriptions by Perrier de la Bâthie (1953a) of Malagasy *Eugenia* were relatively incomplete, and many of his infraspecific names are invalid. Because preliminary studies suggest some of those infraspecific taxa are worthy of recognition, new names will be necessary in future combinations after additional study.

Malagasy *Eugenia* have a relative paucity of easily diagnostic, qualitatively different morphological character states compared to some baccate genera of Myrtaceae (e.g., Snow et al. 2003; Snow 2008), including the related genus *Syzygium*. For example, few species of *Eugenia* have winged or quadrangular branchlets, apically attenuate calyx lobes, costate fruits, or other qualitatively distinct character states that recur in many other baccate genera, and which simplify identifications and the writing of keys (e.g., Snow 2008; Snow et al. 2001, 2008). Although additional undescribed species are known with a high degree of confidence, further research and more collections will be needed to produce a complete taxonomic treatment of the genus for Madagascar.

Despite additional new species of *Eugenia* remaining to be described and other gaps in knowledge of Malagasy taxa, tentative patterns of distribution, ecological preferences and evolution are beginning to emerge (Snow et al. 2014). One: Moderately high levels of specific diversity of *Eugenia* can occur in relatively localized areas, such as the approximately 15 species (including some undescribed) between Taolagnaro and Sainte Luce on the southeast coast. Two: Most *Eugenia* occupy one of three general areas: i) high altitudes in the northern mountainous massifs; ii) humid forests in the vicinity of eastern escarpment; or iii) in drier lowland habitats near the eastern coast. Three: Most species of *Eugenia* in Madagascar have relatively limited geographic ranges. This contrasts with some Malagasy species of *Syzygium* Gaertn., the other genus of Myrtaceae native to Madagascar, which includes some widely distributed species, such as *Syzygium bernieri* (H. Perrier) Labat & Schatz, *Syzygium lububre* (H. Perrier) Labat & Schatz, *Syzygium micropodum* (Baker) Labat & Schatz, *Syzygium parkeri* (Baker) Labat & Schatz, *Syzygium phyillyreifolium* (Baker) Labat & Schatz, *Syzygium sakalavarum* (H. Perrier) Labat & Schatz, and two other species in need of new names (Byng 2014; Byng et al. in press). Given that many species of *Eugenia* are relatively short trees or shrubs, in contrast with many of *Syzygium* that are canopy or emergent species, the average dispersal distance of fruits of *Eugenia* species may be considerably less than *Syzygium*. Four: No species of *Eugenia* is an ecological dominant, although some species can be locally common, such as *Eugenia echinulata* and *Eugenia obovatifolia*. Five: Cauliflory (flowers arising on main bole) and ramiflory (flowers arising on naked branches below leaves) are common methods of floral presentation, and often occur largely in lieu of terminal or axillary presentations. Cauliflory and ramiflory presumably reflect selective pressures for pollination and/or fruit dispersal. Six: All known species have whitish, cream-colored, or pinkish-magenta petals. In contrast, red, yellow, orange, purple or blue petals are unknown. While almost nothing is known about the pollination biology of Malagasy *Eugenia*, the petal colors likely reflect selection pressures of generalized pollinators such as bees. Seven: Selection has sometimes favored relatively large fruits and embryos (e.g., *Eugenia andapae*, *Eugenia manomboensis*, *Eugenia iantarensis*, *Eugenia guillotii*, *Eugenia manomboensis*, *Eugenia ravelonarivoi*), which suggests consumption of and dispersal by mammals or large birds. Lemurs (this paper; Snow et al. 2012) are among the known consumers, and information concerning bird consumption would be appreciated by the first author. Field studies documenting the pollinators of flowers and consumers of fruits of *Eugenia* (and *Syzygium*) would be valuable graduate theses. Eight: Although dioecy in *Eugenia* is well known from western and southern Africa and adjacent areas (van Wyk and Lowry 1988; Bernardini et al. 2014, J. Byng, pers. comm. 2014), this reproductive system has not yet been documented among Madagascar members of *Eugenia*.

Many species of Malagasy *Eugenia*, including several proposed here, are Threatened, Endangered, or Critically Endangered following IUCN Criteria and Recommendations (IUCN 2012). In view of rapidly continuing deforestation in Madagascar, the occurrence of many from five or fewer collections, and that some species are known from only flowering or fruiting material, additional collecting of *Eugenia* should remain a high priority.

We believe that at least ten or more additional species remain to be described, and stress here that more material of other species would enhance our knowledge of taxonomy, distribution, and conservation priorities. Duplicate material of Malagasy *Eugenia* sent to the first author at the T.M. Sperry Herbarium (KSP) for future studies would be greatly appreciated.

**Corrigendum:** The correct collection number for the paratype specimen of *Eugenia vanwykiana* N. Snow is Razakamalala 1234 et al. The protologue incorrectly cited the collection number as 1324 (Snow et al. 2012).

## Supplementary Material

XML Treatment for Eugenia andapae

XML Treatment for Eugenia barriei

XML Treatment for Eugenia bemangidiensis

XML Treatment for Eugenia calciscopulorum

XML Treatment for Eugenia delicatissima

XML Treatment for Eugenia echinulata

XML Treatment for Eugenia gandhii

XML Treatment for Eugenia hazonjia

XML Treatment for Eugenia iantarensis

XML Treatment for Eugenia malcomberi

XML Treatment for Eugenia manomboensis

XML Treatment for Eugenia obovatifolia

XML Treatment for Eugenia ranomafana

XML Treatment for Eugenia ravelonarivoi

XML Treatment for Eugenia razakamalalae

XML Treatment for Eugenia richardii

XML Treatment for Eugenia tiampoka

XML Treatment for Eugenia wilsoniana
